# Variational Deep Alliance: A Generative Auto-Encoding Approach to Longitudinal Data Analysis

**DOI:** 10.3390/e28010113

**Published:** 2026-01-18

**Authors:** Shan Feng, Wenxian Xie, Yufeng Nie

**Affiliations:** School of Mathematics and Statistics, Northwestern Polytechnical University, Xi’an 710129, China; wenxianxie@nwpu.edu.cn

**Keywords:** longitudinal data, Variational Auto-Encoder, deep generative model, marginal model, representation learning, clustering, 68T07, 82M30, 62C10, 62H30, 62M10

## Abstract

Rapid advancements in the field of deep learning have had a profound impact on a wide range of scientific studies. This paper incorporates the power of deep neural networks to learn complex relationships in longitudinal data. The novel generative approach, Variational Deep Alliance (VaDA), is established, where an “alliance” is formed across repeated measurements via the strength of Variational Auto-Encoder. VaDA models the generating process of longitudinal data with a unified and well-structured latent space, allowing outcomes prediction, subjects clustering and representation learning simultaneously. The integrated model can be inferred efficiently within a stochastic Auto-Encoding Variational Bayes framework, which is scalable to large datasets and can accommodate variables of mixed type. Quantitative comparisons to those baseline methods are considered. VaDA shows high robustness and generalization capability across various synthetic scenarios. Moreover, a longitudinal study based on the well-known CelebFaces Attributes dataset is carried out, where we show its usefulness in detecting meaningful latent clusters and generating high-quality face images.

## 1. Introduction

Longitudinal datasets naturally arise in a wide variety of fields and applications, such as social science, biomedicine and agriculture. They contain repeated measurements over multiple time points or spaces, allowing researchers to observe and investigate the temporal and spatial evolution about behaviors and relationships. Different to conventional cross-sectional data, where the sample points are collected independently, the correlated measurements from a subject make longitudinal data analysis a challenging problem. Marginal model [1] focusing on the population-averaged effects is a classical approach for longitudinal data analysis, where consistency inference results can be obtained by solving Generalized Estimating Equations (GEEs) with “working” correlation assumptions [2].

In recent years, rapid advancements in the field of deep learning have had a profound impact in a wide range of scientific studies. The representation learning techniques enable transforming high-dimensional data into their meaningful low-dimensional representations, facilitating a wide variety of downstream tasks hindered by the sheer size and complexity of the original datasets [3]. The Variational Auto-Encoder (VAE) introduced by Kingma and Welling [4] has been extensively used for probabilistic representation learning. It investigates the underlying deep generative model within an efficient stochastic Auto-Encoding Variational Bayes (AEVB) framework.

In this paper, we develop the Variational Deep Alliance (VaDA), a full deep generative model for longitudinal data analysis, where the data of covariates and responses are generated hierarchically from a unified and well-structured latent space. An important observation about the classical marginal model is that it cannot handle complex non-linear effects of covariates and is often hindered by the high-dimensional feature spaces that are intrinsically sparse [5]. In addition, the GEE approach with “working” correlation assumptions mainly models simple covariance structures of measurements, the misspecification of which can damage the estimation efficiency of marginal effects and further influence the prediction accuracy [1]. These are where we can incorporate the power of deep neural networks to learn complex relationships. Specifically, VaDA forms a deep “alliance” across repeated measurements via the strength of VAE, breaking out of the “working” assumptions from the GEEs based marginal modeling approach. From an overall perspective, the auto-encoding in VaDA proceeds in two directions, i.e., cross-sectionally (with X-encoder and X-decoder) and longitudinally (with Y-encoder and Y-decoder), to learn structured latent embeddings and make accurate predictions by probabilistic decoding.

In addition, the subjects in a longitudinal study can originate from subgroups characterized by distinguishable clusters in the latent space. Modeling such heterogeneity is significant in various situations, such as customer segmentation, precision medicine, etc. In VaDA, clustering of subjects is realized by forging it with a mixture model skeleton. The integrated model can be inferred efficiently within a stochastic AEVB framework, which is scalable to large datasets and can accommodate covariates and responses of mixed type. We use synthetic datasets to demonstrate the robustness and generalization capability of VaDA in prediction and clustering. A longitudinal study based on the well-known CelebFaces Attributes (CelebA) dataset [6] in computer vision is carried out, where we show its usefulness in detecting meaningful latent clusters and generating high-quality face images.

## 2. Related Works

### 2.1. Classical Approaches for Longitudinal Data Analysis

Besides the marginal model, the conditional model [7,8] and the mixed effects model [9,10] are two alternative classical approaches for longitudinal data analysis. The conditional model assumes directly a dependence structure between components of repeated measurements and models the joint probability as a multiplication of several transition probabilities. In the mixed effects model, the random subject-specific effects are introduced to induce the dependence relationship between measurements. Generally, these classical approaches are not accessible immediately to high-dimensional datasets. Dimension reduction or feature selection procedures should be conducted for data preprocessing. Moreover, these methods mainly tackle the linear effects of covariates. In [11,12], feature transformations were examined to optimize the non-linear regression relationships.

### 2.2. Deep Learning-Based Longitudinal Data Analysis

Following the efforts of VAE, Sohn et al. [13] proposed the deep conditional VAE approach as a way to incorporate auxiliary information of covariates for representation learning and prediction. However, the standard VAE and the conditional VAE are both designed for i.i.d. observations. In [14], the Gaussian Process Prior VAE (GPPVAE) approach was proposed to account for the association between sample points, which is crucial for correct model specification and optimal inference results in a longitudinal study. GPPVAE replaces the i.i.d. priors over the latent space with a Gaussian process prior, enabling specification of within-subject correlations through covariance functions [15]. It has been adapted for heterogeneous types of covariates [16] and responses [17], and for situations with missing data [18].

While GPPVAE models the temporal and spatial correlations via a covariance function in the latent space, the proposed VaDA approach induces the dependence relationships through a deep generative network. In addition, the covariates introduced in GPPVAE mainly serve as auxiliary information fed into the covariance function and are typically low-dimensional, thus not significantly reducing the inference efficiency. VaDA models responses and covariates in a full generative manner, where the probabilistic auto-encoding networks in two separate but unified directions allow handling response variables and covariates both of high-dimensional. Regarding the data privacy, such an integrated generative model riches uncertainty over the latent space and benefits the out-of-sample data generation essential for data sharing [19]. Moreover, GPPVAE is challenged by handling the cubic computational complexity in the length of repeated measurements, where additional assumptions such as low-rank factorization of the covariance matrix are required to simplify the computation and to reduce the memory burden [14]. In contrast, borrowing the power of deep neural networks, VaDA can handle theoretically arbitrary complex dependence structures in the data.

A separate body of related works for correlated observations structures the latent space of time series by dynamical systems. In [20], the VAE approach was adapted by introducing a deep Kalman filter to the latent representations. Chung et al. [21] and Johnson et al. [22] embedded the VAE into a recurrent neural network for modeling high-dimensional correlated sequences. However, these models are more suitable to time series and incorporating the information of covariates is still an area to be explored.

Recent efforts concatenating the power of deep learning with longitudinal data reassembled the mixed effects model with a feed-forward neural network to account for the complex non-linear effects of covariates [23]. As an extension of the multilayer perceptron regressor, this model is not a full generative model and mainly fitted for prediction purpose.

### 2.3. Clustering of Longitudinal Data

Clustering of longitudinal data remains a relatively under-explored area. The mixture of marginal models [24] introduces an overall mixture model framework to specify the possible heterogeneity of marginal effects in different clusters. The “working” correlation matrix is applied to model the dependence between measurements in each cluster, resulting in a maximization step in the EM algorithm as solving *K* (number of clusters) systems of GEEs. The grouped GEE approach [25] is a distance-based clustering algorithm for longitudinal data. It solves the GEEs in separate groups of subjects and optimizes the grouping using the Magalanobis distance with the estimated “working” correlation matrices. The two steps are implemented iteratively until reaching a stable point. The methods mentioned inherit the limitations of traditional models in handling non-linear effects of covariates and are not suitable for high-dimensional settings.

The Variational Deep Embedding (VaDE) approach [26] is a widely acknowledged variant of VAE for clustering analysis. It specifies a Gaussian mixture prior for the latent variables and is aimed at learning good representations that capture the statistical structure of the data. In a recent study about survival data clustering [27], the Variational Deep Survival Clustering (VaDeSC) approach related the latent representation of feature data from a VaDE encoder with the survival outcome by cluster-specific survival models to allow simultaneous clustering, representation learning and survival prediction [28]. Nevertheless, these approaches do not consider the relationships between sample points and assume i.i.d. observations. In VaDA, we rich the latent space of VaDE with cluster-specific marginal models to realize simultaneous clustering, representation learning and prediction for longitudinal data.

## 3. Methods

In this section, we present VaDA—a generative auto-encoding approach to longitudinal data analysis, which is built on the success of VAE, VaDE and VaDeSC.

### 3.1. Problem Settings

The longitudinal dataset under consideration consists of *N* targeting subjects, such as stocks, patients and varieties of crop. The experimental design includes observing each subject over *L* different time points or locations on some outcome of interest and *P* relevant features, which constitute the observed data for response and covariates. Denote the covariate data of subject n,n=1,2,…,N as $X_{n}={\left(x_{n1},x_{n2},\ldots ,x_{nL}\right)}^{T}$, where $x_{nl}={\left(x_{nl1},x_{nl2},\ldots ,x_{nlP}\right)}^{T}$ is the data at time point or location l,l=1,2,…,L. The response information of subject *n* is denoted as $y_{n}={\left(y_{n1},y_{n2},\ldots ,y_{nL}\right)}^{T}$. In our study, the *P* features can be of mixed type, i.e., $x_{nlp}$ can be real-valued or discrete for p=1,2,…,P. To ease interpretation, we only consider the univariate outcome, which can be real-valued or discrete. Generalization to the multivariate case can be realized within the proposed framework. We leave formal exploration of this part to future work. For clustering of the longitudinal data, we consider for each subject a latent class label $c_{n}$, which is unobserved and takes value in {1, 2,…,K}. *K* is the total number of clusters. The problem then is threefold: (i) to make prediction of the outcome; (ii) to uncover a clustering for the subjects; (iii) to obtain the low-dimensional representations for the feature data.

### 3.2. Generative Process

We assume that the observed data ${\left\{y_{n},X_{n}\right\}}_{n=1}^{N}$ are independently and identically generated from a random process consisting of the following steps.

1. Choose a class label $c_{n}∼Cat\left(\pi \right)$.
2. For l=1,2,…,L:
  (a) Choose a latent vector $z_{nl}∼N\left(\mu_{c_{n}},\mathrm{diag}\left(\sigma_{c_{n}}^{2}\right)\right)$.
  (b) Generate $x_{nl}$ by
    - if $x_{nlp}$ is real-valued, choose $x_{nlp}∼N\left({\tilde{\mu }}_{x,p},{\tilde{\sigma }}_{x,p}^{2}\right)$,
    - if $x_{nlp}$ is categorical, choose $x_{nlp}∼Cat\left({\tilde{\mu }}_{x,p}\right)$,
    where the parameters are computed by
    $$\left\{\left({\tilde{\mu }}_{x,p},{\tilde{\sigma }}_{x,p}^{2}\right),p\in V\left(con\right);\;{\tilde{\mu }}_{x,p},p\in V\left(cat\right)\right\}=f_{x}\left(z_{nl};\theta \right).$$
  (c) Generate latent quantity $u_{nl}∼N\left(\beta_{c_{n},0}+z_{nl}^{T}\beta_{c_{n}},\sigma_{u,c_{n}}^{2}\right)$.
3. Generate $y_{n}$ by
  (a) For real-valued outcome, choose $y_{n}∼N\left({\tilde{\mu }}_{y},\mathrm{diag}\left({\tilde{\sigma }}_{y}^{2}\right)\right)$ with
    $$\left\{{\tilde{\mu }}_{y},{\tilde{\sigma }}_{y}^{2}\right\}=f_{y}\left(u_{n};\phi \right),$$
    where ${\tilde{\mu }}_{y}={\left({\tilde{\mu }}_{y,1},{\tilde{\mu }}_{y,2},\ldots ,{\tilde{\mu }}_{y,L}\right)}^{T}$ and ${\tilde{\sigma }}_{y}^{2}={\left({\tilde{\sigma }}_{y,1}^{2},{\tilde{\sigma }}_{y,2}^{2},\ldots ,{\tilde{\sigma }}_{y,L}^{2}\right)}^{T}$.
  (b) For categorical outcome, choose $y_{n}∼\prod_{l=1}^{L}Cat\left({\tilde{\mu }}_{y,l}\right)$ with
    $${\tilde{\mu }}_{y}=f_{y}\left(u_{n};\phi \right),$$
    where ${\tilde{\mu }}_{y}=\left\{{\tilde{\mu }}_{y,1},{\tilde{\mu }}_{y,2},\ldots ,{\tilde{\mu }}_{y,L}\right\}$.

We use $Cat\left(\tau \right)$ to denote the categorical distribution parameterized by τ, where $\tau ={\left(\tau_{1},\tau_{2},\ldots ,\tau_{G}\right)}^{T}\in R_{+}^{G}$ and $\sum_{g=1}^{G}\tau_{g}=1$. *G* is the number of categories. N(η,Σ) denotes the Gaussian distribution with mean η and covariance Σ. $z_{nl}={\left(z_{nl1},z_{nl2},\ldots ,z_{nlD}\right)}^{T}$ is the latent representation or encoding of $x_{nl}$. *D* is the dimensions of $z_{nl}$ and we have D<P. Denote the data of latent representations as $Z_{n}={\left(z_{n1},z_{n2},\ldots ,z_{nL}\right)}^{T}$. For k=1,2,…,K, $\mu_{k}={\left(\mu_{k,1},\mu_{k,2},\ldots ,\mu_{k,D}\right)}^{T}\in R^{D}$ and $\mathrm{diag}(\sigma_{k}^{2})$, where $\sigma_{k}^{2}={\left(\sigma_{k,1}^{2},\sigma_{k,2}^{2},\ldots ,\sigma_{k,D}^{2}\right)}^{T}\in R_{+}^{D}$ are the mean vector and covariance matrix of $z_{nl}$ in class *k*. Denote $\mu ={\left\{\mu_{k}\right\}}_{k=1}^{K}$ and $\sigma^{2}={\left\{\sigma_{k}^{2}\right\}}_{k=1}^{K}$.

We use V(con) and V(cat) to denote the sets of indexes for real-valued and categorical features, respectively. It has V(con)∩V(cat)=∅ and V(con)∪V(cat)={1,2,…,P}. Collectively, the parameters to generate $x_{nl}$ are derived from a decoding neural network $f_{x}$ with input $z_{nl}$ and parameterized by θ.

The quantities $u_{n}={\left(u_{n1},u_{n2},\ldots ,u_{nL}\right)}^{T}$ are the intermediate latent states of the correlated response variables, which are mutually independent given the class label and structured by the underlying marginal model with cluster-specific marginal mean functions $\beta_{k,0}+z_{nl}^{T}\beta_{k},k=1,2,\ldots ,K$. We assume that $z_{nld},d=1,2,\ldots ,D$ has linear effects on $u_{nl}$ and denote the marginal effects in cluster *k* as $\beta_{k}={\left(\beta_{k,1},\beta_{k,2},\ldots ,\beta_{k,D}\right)}^{T}\in R^{D}$ and residual variance as $\sigma_{u,k}^{2}\in R_{+}$. $\beta_{k,0}\in R$ allows modeling the intercept of the marginal mean function. Denote $\beta ={\left\{\beta_{k,0},\beta_{k}\right\}}_{k=1}^{K}$ and $\sigma_{u}^{2}={\left\{\sigma_{u,k}^{2}\right\}}_{k=1}^{K}$. $u_{n}$ has the practical meaning as “pure outcomes”.

**Figure 1:**
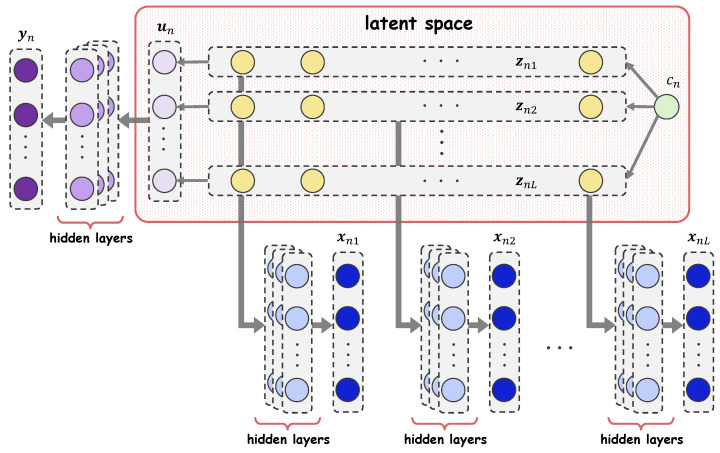
Data generating process of VaDA.

The final observed outcomes $y_{n}$ are produced through a non-observable mixing process, e.g., the gene–environment interaction in bioscience and the temporal and spatial evolution of behavior. We simulate this process artificially using the decoder $f_{y}$ which has input $u_{n}$ and is parameterized by ϕ. Modeling this process is essential in VaDA, where the measurements within a subject henceforth enter into a deep “alliance”. The overall generative process assumed by VaDA is depicted in Figure 1.

According to the generative process described above, the complete-data likelihood $p(y_{n},u_{n},X_{n},Z_{n},c_{n})$ can be factorized as

(1) $$p\left(y_{n},u_{n},X_{n},Z_{n},c_{n}\right)=p\left(y_{n}|u_{n}\right)p\left(u_{n}|Z_{n},c_{n}\right)p\left(X_{n}|Z_{n}\right)p\left(Z_{n}|c_{n}\right)p\left(c_{n}\right),$$

which can be further factorized by

$$\begin{array}{cc} \; & p\left(u_{n}|Z_{n},c_{n}\right)=\prod_{l=1}^{L}p\left(u_{nl}|z_{nl},c_{n}\right),\\ \; & p\left(X_{n}|Z_{n}\right)=\prod_{l=1}^{L}p\left(x_{nl}|z_{nl}\right),\\ \; & p\left(Z_{n}|c_{n}\right)=\prod_{l=1}^{L}p\left(z_{nl}|c_{n}\right). \end{array}$$

Taking the real-valued outcome as an example, the probabilities composing (1) are defined by

$$\begin{array}{cc} \; & p\left(c_{n}\right)=Cat\left(c_{n};\pi \right),\\ \; & p\left(z_{nl}|c_{n}\right)=N\left(z_{nl};\mu_{c_{n}},\mathrm{diag}\left(\sigma_{c_{n}}^{2}\right)\right),\\ \; & p\left(x_{nl}|z_{nl}\right)=\prod_{p\in V(con)}N\left(x_{nlp};{\tilde{\mu }}_{x,p},{\tilde{\sigma }}_{x,p}^{2}\right)\prod_{p\in V(cat)}Cat\left(x_{nlp};{\tilde{\mu }}_{x,p}\right),\\ \; & p\left(u_{nl}|z_{nl},c_{n}\right)=N\left(u_{nl};\beta_{c_{n},0}+z_{nl}^{T}\beta_{c_{n}},\sigma_{u,c_{n}}^{2}\right),\\ \; & p\left(y_{n}|u_{n}\right)=N\left(y_{n};{\tilde{\mu }}_{y},\mathrm{diag}\left({\tilde{\sigma }}_{y}^{2}\right)\right). \end{array}$$

### 3.3. Variational Lower Bound

Based on the complete-data likelihood given in (1), the observed-data log-likelihood of VaDA can be written as

$$\log p\left(y_{n},X_{n}\right)=\log\;\int \int \;\sum_{c_{n}}p\left(y_{n}|u_{n}\right)p\left(u_{n}|Z_{n},c_{n}\right)p\left(X_{n}|Z_{n}\right)p\left(Z_{n}|c_{n}\right)p\left(c_{n}\right)\;dZ_{n}\;du_{n}.$$

By Jensen’s inequality, it follows

$$\log p\left(y_{n},X_{n}\right)\geq E_{q(u_{n},Z_{n},c_{n}|y_{n},X_{n})}\left[\log\frac{p\left(y_{n}|u_{n}\right)p\left(u_{n}|Z_{n},c_{n}\right)p\left(X_{n}|Z_{n}\right)p\left(Z_{n}|c_{n}\right)p\left(c_{n}\right)}{q(u_{n},Z_{n},c_{n}|y_{n},X_{n})}\right]=:L_{\mathrm{ELBO}}\left(y_{n},X_{n}\right),$$

where $L_{\mathrm{ELBO}}\left(y_{n},X_{n}\right)$ is the evidence lower bound objective (ELBO) of the log-likelihood $\log p(y_{n},X_{n})$, and $q(u_{n},Z_{n},c_{n}|y_{n},X_{n})$ is the variational auxiliary function to approximate the inaccessible true posterior $p(u_{n},Z_{n},c_{n}|y_{n},X_{n})$. In VaDA, it is assumed that the auxiliary $q(u_{n},Z_{n},c_{n}|y_{n},X_{n})$ is a mean-field distribution and can be factorized as

(2) $$q\left(u_{n},Z_{n},c_{n}|y_{n},X_{n}\right)=q\left(u_{n}|y_{n}\right)q\left(Z_{n}|X_{n}\right)q\left(c_{n}|y_{n},X_{n}\right),$$

where

$$q\left(Z_{n}|X_{n}\right)=\prod_{l=1}^{L}q\left(z_{nl}|x_{nl}\right).$$

Under some mathematical manipulations, it follows that the lower bound $L_{\mathrm{ELBO}}\left(y_{n},X_{n}\right)$ can be written as

(3) $$\begin{array}{cc} L_{\mathrm{ELBO}}\left(y_{n},X_{n}\right) & =E[\log p\left(y_{n}|u_{n}\right)+\log p\left(u_{n}|Z_{n},c_{n}\right)+\log p\left(X_{n}|Z_{n}\right)+\log p\left(Z_{n}|c_{n}\right)+\log p\left(c_{n}\right)\\ \; & \;-\log q\left(u_{n}|y_{n}\right)-\log q\left(Z_{n}|X_{n}\right)-\log q\left(c_{n}|y_{n},X_{n}\right)], \end{array}$$

where the expectation is taken w.r.t. $q(u_{n},Z_{n},c_{n}|y_{n},X_{n})$ with factorization (2).

We use neural networks to model $q(u_{n}|y_{n})$ and $q(z_{nl}|x_{nl})$, i.e.,

(4) $$q\left(u_{n}|y_{n}\right)=N\left(u_{n};{\tilde{\mu }}_{u},\mathrm{diag}\left({\tilde{\sigma }}_{u}^{2}\right)\right),\;\left\{{\tilde{\mu }}_{u},{\tilde{\sigma }}_{u}^{2}\right\}=g_{u}\left(y_{n};\lambda \right),$$

where ${\tilde{\mu }}_{u}={\left({\tilde{\mu }}_{u,1},{\tilde{\mu }}_{u,2},\ldots ,{\tilde{\mu }}_{u,L}\right)}^{T}$ and ${\tilde{\sigma }}_{u}^{2}={\left({\tilde{\sigma }}_{u,1}^{2},{\tilde{\sigma }}_{u,2}^{2},\ldots ,{\tilde{\sigma }}_{u,L}^{2}\right)}^{T}$ are parameters for the posterior density function of $u_{n}$. They are derived from the encoder network $g_{u}$ with input $y_{n}$ and parameters λ. Similarly,

(5) $$\begin{array}{c} q\left(z_{nl}|x_{nl}\right)=N\left(z_{nl};{\tilde{\mu }}_{z},\mathrm{diag}\left({\tilde{\sigma }}_{z}^{2}\right)\right),\;\left\{{\tilde{\mu }}_{z},{\tilde{\sigma }}_{z}^{2}\right\}=g_{z}\left(x_{nl};\gamma \right), \end{array}$$

where ${\tilde{\mu }}_{z}={\left({\tilde{\mu }}_{z,1},{\tilde{\mu }}_{z,2},\ldots ,{\tilde{\mu }}_{z,D}\right)}^{T}$ and ${\tilde{\sigma }}_{z}^{2}={\left({\tilde{\sigma }}_{z,1}^{2},{\tilde{\sigma }}_{z,2}^{2},\ldots ,{\tilde{\sigma }}_{z,D}^{2}\right)}^{T}$ are parameters for the posterior distribution of $z_{nl}$. They are derived from the encoder network $g_{z}$ with input $x_{nl}$ and parameters γ.

We do not introduce excessive assumptions for distribution $q(c_{n}|y_{n},X_{n})$ but obtain it by integrating $u_{n}$ and $Z_{n}$ from the accessible posterior $p(c_{n}|u_{n},Z_{n})$, i.e.,

$$q\left(c_{n}|y_{n},X_{n}\right)=E_{q\left(u_{n}|y_{n}\right)q\left(Z_{n}|X_{n}\right)}\left[p(c_{n}|u_{n},Z_{n})\right],$$

where

$$p\left(c_{n}|u_{n},Z_{n}\right)=\frac{p\left(u_{n}|Z_{n},c_{n}\right)p\left(Z_{n}|c_{n}\right)p\left(c_{n}\right)}{\sum_{c_{n}}p\left(u_{n}|Z_{n},c_{n}\right)p\left(Z_{n}|c_{n}\right)p\left(c_{n}\right)}=:h\left(u_{n},Z_{n},c_{n};H\right).$$

Therefore, $h(u_{n},Z_{n},c_{n};H)$ is a known function with inputs $\left(u_{n},Z_{n},c_{n}\right)$ and parameters $H=\left\{\beta ,\sigma_{u}^{2},\mu ,\sigma^{2},\pi \right\}$.

### 3.4. Understanding the ELBO of VaDA

The ELBO in (3) can be rearranged as

$$L_{\mathrm{ELBO}}\left(y_{n},X_{n}\right)=E_{q\left(u_{n}|y_{n}\right)q\left(Z_{n}|X_{n}\right)}\left[\log p(y_{n},X_{n}|u_{n},Z_{n})\right]-D_{\mathrm{KL}}\left[q\left(u_{n},Z_{n},c_{n}|y_{n},X_{n}\right)\left|\right|p\left(u_{n},Z_{n},c_{n}\right)\right].$$

The first term is the reconstruction term, which encourages VaDA to explain the dataset well. It can be further separated into two parts as

$$E_{q\left(u_{n}|y_{n}\right)q\left(Z_{n}|X_{n}\right)}\left[\log p(y_{n},X_{n}|u_{n},Z_{n})\right]=E_{q(u_{n}|y_{n})}\left[\log p(y_{n}|u_{n})\right]+\sum_{l=1}^{L}E_{q(z_{nl}|x_{nl})}\left[\log p(x_{nl}|z_{nl})\right],$$

corresponding to the reconstructions for outcomes and feature data, respectively. The second term is the Kullback-Leibler (KL) divergence from the prior of $\left(u_{n},Z_{n},c_{n}\right)$ to their auxiliary posterior, which therefore regularizes the latent embedding $\left(u_{n},Z_{n}\right)$ to lie on a structured manifold as a mixture model. Appendix B illustrates the evolution of model structures and the induced changes of ELBOs between VAE, VaDE and VaDA.

Due to the complexity of the decoding and encoding networks, the expectation for the reconstruction term cannot be computed exactly. As in VAE and VaDE, the Stochastic Gradient Variational Bayes (SGVB) estimator and the reparameterization trick [4] are applied, which gives

$$E_{q(u_{n}|y_{n})}\left[\log p(y_{n}|u_{n})\right]\approx \frac{1}{M}\sum_{m=1}^{M}\log N\left(y_{n};{\tilde{\mu }}_{y}^{\left(m\right)},\mathrm{diag}\left({\tilde{\sigma }}_{y}^{2,(m)}\right)\right),$$

and

$$E_{q(z_{nl}|x_{nl})}\left[\log p(x_{nl}|z_{nl})\right]\approx \frac{1}{M}\sum_{m=1}^{M}\left[\sum_{p\in V(con)}\log N\left(x_{nlp};{\tilde{\mu }}_{x,p}^{\left(m\right)},{\tilde{\sigma }}_{x,p}^{2,(m)}\right)+\sum_{p\in V(cat)}\log Cat\left(x_{nlp};{\tilde{\mu }}_{x,p}^{\left(m\right)}\right)\right].$$

We compute ${\tilde{\mu }}_{y}^{\left(m\right)}$ and ${\tilde{\sigma }}_{y}^{2,(m)}$ as

$$\left\{{\tilde{\mu }}_{y}^{\left(m\right)},{\tilde{\sigma }}_{y}^{2,(m)}\right\}=f_{y}\left(u_{n}^{\left(m\right)};\phi \right),$$

where $u_{n}^{\left(m\right)}$ is the *m*th sample from $q(u_{n}|y_{n})$ to produce the Monte Carlo (MC) approximation and can be obtained by

$$u_{n}^{\left(m\right)}={\tilde{\mu }}_{u}+{\tilde{\sigma }}_{u}\cdot \epsilon_{u}^{\left(m\right)},\;\epsilon_{u}^{\left(m\right)}={\left(\epsilon_{u,1}^{\left(m\right)},\epsilon_{u,2}^{\left(m\right)},\ldots ,\epsilon_{u,L}^{\left(m\right)}\right)}^{T}\;\mathrm{and}\;\epsilon_{u,l}^{\left(m\right)}∼N\left(0,1\right).$$

${\tilde{\mu }}_{u}$ and ${\tilde{\sigma }}_{u}$ are obtained from the encoding network $g_{u}$ in (4). Meanwhile,

$$\left\{\left({\tilde{\mu }}_{x,p}^{\left(m\right)},{\tilde{\sigma }}_{x,p}^{2,(m)}\right),p\in V\left(con\right);\;{\tilde{\mu }}_{x,p}^{\left(m\right)},p\in V\left(cat\right)\right\}=f_{x}\left(z_{nl}^{\left(m\right)};\theta \right),$$

where $z_{nl}^{\left(m\right)}$ is the *m*th sample from $q(z_{nl}|x_{nl})$ generated by

$$z_{nl}^{\left(m\right)}={\tilde{\mu }}_{z}+{\tilde{\sigma }}_{z}\cdot \epsilon_{z}^{\left(m\right)},\;\epsilon_{z}^{\left(m\right)}={\left(\epsilon_{z,1}^{\left(m\right)},\epsilon_{z,2}^{\left(m\right)},\ldots ,\epsilon_{z,D}^{\left(m\right)}\right)}^{T}\;\mathrm{and}\;\epsilon_{z,d}^{\left(m\right)}∼N\left(0,1\right).$$

${\tilde{\mu }}_{z}$ and ${\tilde{\sigma }}_{z}$ are obtained from network $g_{z}$ in (5). Denote $Z_{n}^{\left(m\right)}={\left(z_{n1}^{\left(m\right)},z_{n2}^{\left(m\right)},\ldots ,z_{nL}^{\left(m\right)}\right)}^{T}$.

Given the MC samples of $u_{n}$ and $Z_{n}$, we can also form an approximation for $q(c_{n}|y_{n},X_{n})$ as

(6) $$q\left(c_{n}=k|y_{n},X_{n}\right)\approx \frac{1}{M}\sum_{m=1}^{M}p\left(c_{n}=k|u_{n}^{\left(m\right)},Z_{n}^{\left(m\right)}\right)=\frac{1}{M}\sum_{m=1}^{M}h\left(u_{n}^{\left(m\right)},Z_{n}^{\left(m\right)},k;H\right)=:{\tilde{\pi }}_{k},$$

for k=1,2,…,K, under which the KL divergence between $q(u_{n},Z_{n},c_{n}|y_{n},X_{n})$ and $p(u_{n},Z_{n},c_{n})$ can be computed analytically (see Appendix A for detailed derivations), given by

$$\begin{array}{cc} D_{\mathrm{KL}} & \left[q\left(u_{n},Z_{n},c_{n}|y_{n},X_{n}\right)\left|\right|p\left(u_{n},Z_{n},c_{n}\right)\right]\\ \; & =\frac{1}{2}\sum_{k=1}^{K}{\tilde{\pi }}_{k}\sum_{l=1}^{L}\left\{\log\sigma_{u,k}^{2}+{\left(\sigma_{u,k}^{2}\right)}^{-1}\left[{\left({\tilde{\mu }}_{u,l}-\beta_{k,0}-{\tilde{\mu }}_{z}^{T}\beta_{k}\right)}^{2}+{\tilde{\sigma }}_{u,l}^{2}+\beta_{k}^{T}\mathrm{diag}\left({\tilde{\sigma }}_{z}^{2}\right)\beta_{k}\right]\right\}\\ \; & \;+\frac{1}{2}\sum_{k=1}^{K}{\tilde{\pi }}_{k}\sum_{l=1}^{L}\sum_{d=1}^{D}\left\{\log\sigma_{k,d}^{2}+{\left(\sigma_{k,d}^{2}\right)}^{-1}\left[{\left({\tilde{\mu }}_{z,d}-\mu_{k,d}\right)}^{2}+{\tilde{\sigma }}_{z,d}^{2}\right]\right\}-\sum_{k=1}^{K}{\tilde{\pi }}_{k}\log\pi_{k}\\ \; & \;-\frac{1}{2}\sum_{l=1}^{L}\log{\tilde{\sigma }}_{u,l}^{2}-\frac{1}{2}\sum_{l=1}^{L}\sum_{d=1}^{D}\log{\tilde{\sigma }}_{z,d}^{2}+\sum_{k=1}^{K}{\tilde{\pi }}_{k}\log{\tilde{\pi }}_{k}. \end{array}$$

It is noticeable that the overall inference framework conducts decoding and encoding processes in two separate directions. The first is the cross-sectional direction from the latent space to the feature space with encoder $g_{z}$ and decoder $f_{x}$, which is the same as for VAE and VaDE. The other is the longitudinal or allying direction from the latent space to the outcomes with encoder $g_{u}$ and decoder $f_{y}$. To ease interpretation, we term $g_{z}$ and $f_{x}$ as X-encoder and X-decoder, and term $g_{u}$ and $f_{y}$ as Y-encoder and Y-decoder.

### 3.5. Clustering and Prediction

In the inference framework of VaDA, clustering of subjects can be realized in two paths. The first is the complete-data case where besides the feature data $X_{n}$ we have already observed the outcomes $y_{n}$. The clustering therefore can be obtained by assigning subject *n* to cluster

(7) $$k^{*}=\underset{k\in \{1,2,\ldots ,K\}}{arg max}q\left(c_{n}=k|y_{n},X_{n}\right),$$

where the probability $q(c_{n}=k|y_{n},X_{n})$ can be approximated by ${\tilde{\pi }}_{k}$ defined in (6). The second path is when the outcomes $y_{n}$ have not been observed and are targets to be predicted. The clustering of subject *n* is given by

(8) $$k^{*}=\underset{k\in \{1,2,\ldots ,K\}}{arg max}q\left(c_{n}=k|X_{n}\right),$$

where

$$q\left(c_{n}=k|X_{n}\right)\approx \frac{1}{M}\sum_{m=1}^{M}p\left(c_{n}=k|Z_{n}^{\left(m\right)}\right)=\frac{1}{M}\sum_{m=1}^{M}\frac{p\left(Z_{n}^{\left(m\right)}|c_{n}=k\right)p\left(c_{n}=k\right)}{\sum_{k=1}^{K}p\left(Z_{n}^{\left(m\right)}|c_{n}=k\right)p\left(c_{n}=k\right)}.$$

Prediction of $y_{n}$ from the observed data $X_{n}$ can be obtained by the following steps. The first step is to transform the feature data $X_{n}={\left(x_{n1},x_{n2},\ldots ,x_{nL}\right)}^{T}$ to their latent representations ${\hat{Z}}_{n}={\left({\hat{z}}_{n1},{\hat{z}}_{n2},\ldots ,{\hat{z}}_{nL}\right)}^{T}$ through the learned X-encoder. We have ${\hat{z}}_{nl}={\tilde{\mu }}_{z}$, which is the output of $g_{z}\left(x_{nl};\gamma \right)$. In the second step, the representations are inserted into the *K* latent cluster-specific marginal models to obtain the estimated pure outcomes ${\hat{u}}_{n}^{k}={\left({\hat{u}}_{n1}^{k},{\hat{u}}_{n2}^{k},\ldots ,{\hat{u}}_{nL}^{k}\right)}^{T},k=1,2,\ldots ,K$, where ${\hat{u}}_{nl}^{k}=\beta_{k,0}+{\hat{z}}_{nl}^{T}\beta_{k}$. Then, we use the Y-decoder to decode ${\hat{u}}_{n}^{k}$ back to the outcomes ${\hat{y}}_{n}^{k}={\hat{\mu }}_{y}^{k}$, which are the outputs of $f_{y}\left({\hat{u}}_{n}^{k};\phi \right)$. The final prediction can be obtained by

$${\hat{y}}_{n}=\sum_{k=1}^{K}q\left(c_{n}=k|X_{n}\right)\cdot {\hat{y}}_{n}^{k},$$

where we have used the probability mass function $q(c_{n}|X_{n})$ not $q(c_{n}|y_{n},X_{n})$ as $y_{n}$ are unobserved.

## 4. Synthetic Study

In this section, we assess the proposed method for longitudinal data analysis through different simulated data scenarios. The objective is to evaluate the quality of clustering and prediction performance obtained coherently using VaDA. The baseline methods include VaDE+MM, VAE+GMM+MM and GMM+MM. VaDE+MM for longitudinal data analysis fits cluster-specific marginal models (MM) based on the representations of feature data and clustering derived simultaneously from VaDE. In VAE+GMM+MM, the clustering is realized by fitting a Gaussian Mixture Model (GMM) based on the obtained representations from VAE. Then, the cluster-specific marginal models are fitted for longitudinal prediction. GMM+MM applies the GMM for clustering and fits the cluster-specific marginal models based on the original dataset.

When fitting marginal models by the GEE approach, three types of “working” correlation assumption were considered, i.e., independence (MM0), ar1 (MM1) and exchangeable (MM2), with increased complexity on the allowed correlation structures [2]. Note that the assumptions MM1 and MM2 are equivalent when the length of measurements L = 2. Another problem is that the clustering results either from VaDE or GMM may differ within subject. To obtain a valid clustering for longitudinal data, we made subtle modifications in the algorithms of VaDE and GMM, where the clustering results for each subject were merged across measurements by summarizing the probability of belonging in each cluster and assigning the subject to cluster having the highest total probability. Due to the frequently appearing “rank deficiency” problem in the marginal models [29], we used the Principal Component Analysis (PCA) [30,31] to project further the feature embeddings in VaDE+MM and VAE+GMM+MM and the original feature data in GMM+MM to orthogonal principal components before fitting the cluster-specific marginal models to ensure smooth implementation of these baselines. The detailed instructions are presented in Appendix G.

Four synthetic scenarios were considered. The generated feature data had dimensions P = 100 and the length of measurements for a subject was L = 4. We used a number of N = 2500 subjects. Therefore, there were totally N × L =  10,000 observations. In Scenarios 11 and 01, we considered the data generated from three clusters with ten-dimensional latent embeddings, where the marginal effects were non-linear. Scenario 11 corresponded to the well-specified case where the data were generated using the VaDA decoders in two separate directions. Scenario 01 used only the X-decoder to generate the feature data and the outcome variables for each subject were mutually independent given by the pure outcomes. In Scenarios 10 and 00, we considered the data generated from three clusters with linear cluster-specific marginal effects. Scenario 10 used only the Y-decoder to induce dependence between outcomes and the feature data were generated directly from a mixture model with diagonal component covariance matrices. In Scenario 00, we considered the baseline where the data were generated following a simple linear regression model in each cluster where neither the X-decoder nor the Y-decoder was applied. See Appendix C for detailed generating processes and parameter configurations in the four scenarios. Here, we consider the real-valued outcome. The categorical case is examined in the following section with the CelebA dataset. In each scenario, 30 random datasets were generated independently to make reliable assessment on the methods.

The deep learning methods VaDA, VaDE and VAE were implemented under the backbone of TensorFlow [32] and trained with the Adam method for optimization [33] at the default learning rate of 0.001. Due to the difference between the inference frameworks, the batch size for VaDA was set as 128, and for VaDE and VAE it was set as 128 × L = 512. For all the three models, the number of training epochs was set as 500, which had been decided approximately by cross-validation (Appendix E). To make a fair comparison, the decoder and encoder network architectures in VaDE and VAE were set the same as the X-decoder and X-encoder in VaDA, respectively. See Appendix D for specific network configurations. We used M = 1 MC sample for the SGVB estimator as its usual settings. Further explorations on this hyperparameter are provided in Appendix F. And opposed to the practice in many deep unsupervised learning methods, we did not pretrain the networks. But a re-scaling of the response and covariate variables to zero-mean and unit-variance was conducted before training the models. The training/testing ratio of the data was set as 4/1. As the latent dimensions *D* and the number of clusters *K* were unknown a priori, we varied the settings of D∈{2,5,10,20,100} and K∈{2,3,4} when fitting each model, which were reasonable quantities considered in practice.

Comparison between methods was based on the metrics of mean square error (MSE) and adjusted Rand index (ARI) [27]. The metric MSE was used to assess the accuracy of prediction, while ARI was computed to evaluate the quality of clustering. As we know the data generating process, the ground-truth class labels can be used post hoc to unambiguously assess the clustering performance. ARI allows evaluation between two data classifications with different number of classes and is not affected by the label switching problem [34].

Table 1 compares the results of MSE on the testing datasets when setting K = 3 in the four scenarios. The fitting results on the training datasets are presented in the Supplementary Materials (Section S1) as a reference. As can be seen, VaDA significantly outperforms the other methods exhibiting high generalization performance across the four scenarios. It is noticeable that VaDA has a remarkably higher performance in Scenarios 11 and 10 (having within-subject correlations), which implies the importance of correctly modeling the within-subject correlations. VaDA can simulate more sophisticated dependence relationships compared with the marginal model with commonly used “working” correlation structures. Additionally, a decreasing on the MSE can be observed in Scenarios 11 and 01 (with non-linear marginal effects) as the dimensions *D* of latent representation increases. While small *D* value could cause a loss of information, larger *D* allows capturing more latent features that improve the prediction accuracy. In our experiments on VaDA, increasing the latent dimensions *D* has not resulted in an over-fitting problem, which however becomes severe when using the methods VaDE+MM and VAE+GMM+MM in Scenarios 10 and 00 (with linear marginal effects). In Scenarios 01 and 00 (no within-subject correlation), VaDA performs slightly inferior to the baseline method GMM+MM. This bias could be a result of overly modeling the within-subject correlations which however do not exist.

Table 2 compares the results of MSE on the testing datasets when setting D = 10 in the four scenarios. We consider the settings K∈{2, 3, 4} while the true number of latent clusters is 3. By an overall inspection, it can be found that the prediction results are not very sensitive to the specification of *K*. VaDA shows stable performance with varied *K* values across the four scenarios and significantly outperforms the other methods in Scenarios 11 and 10 where the within-subject correlations exist. In addition, the baseline method GMM+MM can achieve a good prediction accuracy in Scenarios 01 and 00 (no within-subject correlation). However, its performance degrades heavily in Scenarios 11 and 10 when dependence relationships between outcomes are present. Overall, modeling of the within-subject correlations has a more significant impact on the prediction results in our experiments. The unexpected good performance of GMM+MM in Scenario 01 with non-linear marginal effects could be a result of adequate sample size and no severe rank-deficiency problem in the design matrix transformed by PCA.

The clustering results on the testing datasets assessed by the ARI value are provided in Table 3 and Table 4. The values of ARI closer to one indicate higher degree of matching between two classifications. There are totally five different clustering results under comparison provided by VaDA-YX, VaDA-X, VaDE, VAE+GMM and GMM, respectively. We denote clustering rule (7) of VaDA under complete data as VaDA-YX, and rule (8) with unobserved outcomes as VaDA-X. In Table 3, we fix on K = 3 and test the clustering performance with varied latent dimensions *D*. As can be seen, VaDA-YX and VaDA-X show great robustness in retrieving the ground-truth clusters in all the four scenarios. Even with insufficient latent dimensions *D*, the methods can provide clustering results matching well with the original grouping of the subjects. It is noticeable that there is little difference between the clustering results of VaDA-YX and VaDA-X, which is in line with expectation. In principle, the clustering rules VaDA-YX and VaDA-X differ by an integral with respect to the predictive distribution $p(y_{n}|X_{n})$. If $p(y_{n}|X_{n})$ peaks at the true value of $y_{n}$, the two clustering rules will probabilistically give the same results. The architecture of VaDA can incorporate the diversity of different data generating mechanisms in the four synthetic scenarios and has obtained high prediction accuracy as shown in Table 1 and Table 2. The combined results imply that the overall AEVB framework of VaDA could provide a good approximation for the true predictive distribution, which gives consistent estimators for the outcomes.

The clustering quality of VaDE deteriorates considerably as the latent dimensions *D* is reduced in Scenarios 11 and 01 (with non-linear marginal effects). Comparison between the results of VaDA and VaDE highlights the expressiveness provided by jointly modeling the marginal regression and clustering in the latent space. The VaDA approach exploits the interactions between the covariate data and the response data, which could calibrate the clustering bias with inadequate covariate information. The representations learned by VAE improve the clustering from GMM in Scenarios 11 and 01 (with non-linear marginal effects), but degrade the performance in Scenarios 10 and 00 (with linear marginal effects).

In Table 4, the clustering performances with varied *K* values when fixing on D = 10 are compared. Again, the approaches VaDA-YX and VaDA-X show better generalization performance compared with the other methods. It is noticeable that there is a dramatic decreasing on the ARI value when *K* is smaller than the ground-truth. This observation is conformable with the property of ARI as two subjects originally separated in two classes now could have to be inserted into one cluster. Generally, the performances of VAE+GMM and GMM arrive their optima at the ground-truth *K* value.

Overall, VaDA can achieve superior clustering performance while hardly sacrificing the prediction accuracy. It is robust and shows high generalization performance in those misspecified cases, which provides us with great confidence in applying the VaDA approach for longitudinal data analysis in those real-world settings.

## 5. Application on CelebA Dataset

In this section, we consider a novel application of the proposed method on the CelebA dataset. CelebA is a large-scale face attribute dataset containing 202,599 face images from 10,177 celebrities. The 40 binary attributes annotations per image provide rich source of variability to establish a comprehensive longitudinal study.

We focus on the recognition task on whether a celebrity wears sunglasses from the face image, which constitutes a supervised learning problem with a binary response variable. To form a longitudinal dataset, two face images for each celebrity were randomly chosen: one was wearing sunglasses and the other was not (the order was shuffled). Therefore, there exists apparent within-subject dependence. It can be expected that correctly modeling the relationship between the outcomes of a subject will enhance the performance of representation learning, clustering and image generating. There were totally 3169 celebrities (1199 female celebrities and 1970 male celebrities) who had both the face images with sunglasses and without sunglasses. We used the images from 1200 male celebrities to balance the gender ratio. Finally, the number of subjects in the longitudinal study was N = 2399, the length of measurements was L = 2 and there were totally N × L = 4798 observations. The feature data were organized by cropping and scaling the corresponding face images to 64 × 64 pixels as in [35], which therefore had an input tensor shape (64, 64, 3).

The methods VaDA, VaDE+MM and VAE+GMM+MM, which can realize prediction, clustering and representation learning, were implemented. We did not consider the baseline method GMM+MM here as it can hardly be implemented with efficiency when the data is high-dimensional. While VaDE and VAE inspect the face images in the CelebA longitudinal dataset as fully independent samples, the VaDA method treats the paired image data. As we considered a binary outcome, the link function in the marginal models for VaDE+MM and VAE+GMM+MM was set as “logit” [29]. We also changed the reconstruction term about $y_{n}$ in the ELBO of VaDA to the negative logistic loss function (cross-entropy) [36] to match with the type of outcome. As the values for the latent dimensions *D* and the number of clusters *K* were unknown a priori, we took experiments on D∈{64, 128, 256, 512} and K∈{2, 3, 4}, which were conventional settings. The training/testing ratio was set as 4/1. Again, we used the Adam method for optimization with a learning rate 0.0005. The batch size for VaDA was set as 128 and for VaDE and VAE it was set as 128×L=256. For all the three methods, the number of training epochs was set as 100. To accelerate the progress of training, we used the off-the-shelf network architectures from the improved VAE model in [35] for the X-decoder and X-encoder, which had been used to process the CelebA dataset and achieved good performance in representation learning. Appendix D provides details for the network architectures. Again, no pretraining was conducted. Image data were scaled into [0, 1] by dividing the maximum pixel value 255.

### 5.1. Prediction

Table 5 compares the prediction accuracy of different methods on the training/testing dataset, evaluated through the value of classification error rate. VaDA shows a high generalization performance with various settings of *D* and *K*. It fits well to the training data while making even better prediction on the testing data. Oppositely, VaDE+MM and VAE+GMM+MM show a sign of over-fitting with large *D* values. Specifically, the prediction accuracy of VaDE+MM or VAE+GMM+MM is gradually improved on the training data but worsen on the testing data as the assumed latent dimensions *D* increases. In addition, changing the “working” correlation assumption in the two methods does not save the performance. They even perform better when the independence assumption (MM0) is made. Through the following experiments, we will see that the deteriorated performance could largely come from fitting the cluster-specific marginal models, which are specified and fitted separately from the representation learning stage in VaDE+MM and VAE+GMM+MM.

### 5.2. Clustering

When evaluating the clustering results of CelebA data, we have no prior known subject label to match with as what we have done in the synthetic experiments. Recently, a study demonstrates that visual concepts such as face pose and gender could be manipulated by simple vector arithmetic in the latent space, giving rise to the definition of concept vector for image editing [37]. This strategy provides us a way to visually investigate properties of different clusters by identifying the concept vector from one cluster to another. Specifically, we encode each observed image $x_{nl}$ to its latent representation ${\hat{z}}_{nl}$ through the X-encoder and take averaging over the latent representations within each cluster. Denote the obtained latent vector in cluster *k* as ${\bar{z}}_{k}$. Then, the concept vector from cluster $k^{\prime }$ to *k* can be defined by ${\bar{z}}_{k}-{\bar{z}}_{k^{\prime }}$, which indicates a direction in the latent space encoding certain variations in the image data from cluster $k^{\prime }$ to *k*. If we map ${\hat{z}}_{nl}+\alpha \left({\bar{z}}_{k}-{\bar{z}}_{k^{\prime }}\right)$ back to the image space through the X-decoder, an increasing of the value α will edit the image $x_{nl}$ following the direction from cluster $k^{\prime }$ to *k*. While clustering results of high quality can provide meaningful concept vectors, good generalization performance of X-encoder and X-decoder is also necessary to ensure a satisfactory manifestation of the editing effects.

**Figure 2:**
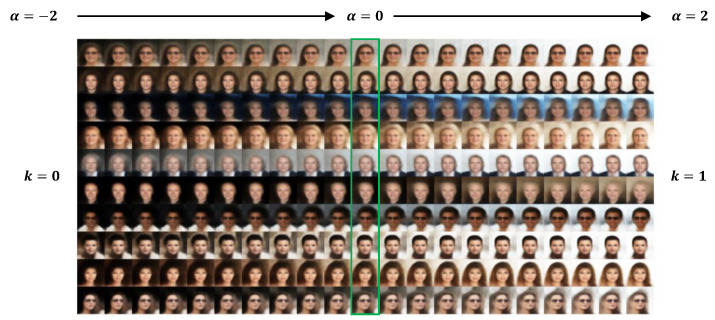
Image editing by concept vector between each pair of clusters based on the results of VaDA with K = 2. The gradient α takes value in [−2, 2]. The results at α = 0 (enclosed by green box) correspond to the reconstruction of original images.

**Figure 3:**
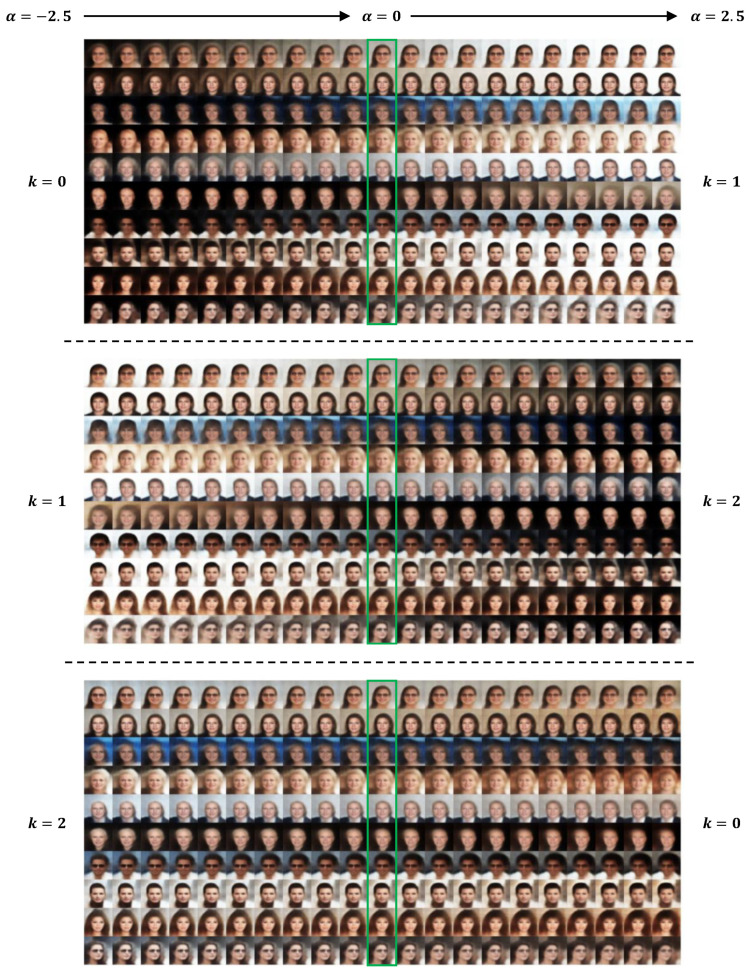
Image editing by concept vector between each pair of clusters based on the results of VaDA with K = 3. The gradient α takes value in [−2.5, 2.5]. The results at α = 0 (enclosed by green box) correspond to the reconstruction of original images.

**Figure 4:**
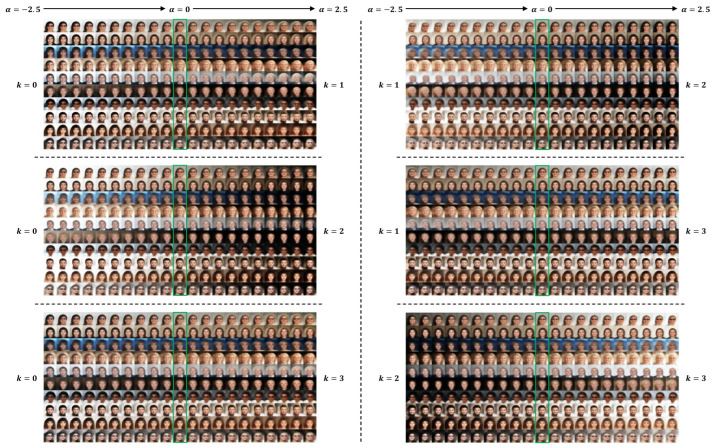
Image editing by concept vector between each pair of clusters based on the results of VaDA with K = 4. The gradient α takes value in [−2.5, 2.5]. The results at α = 0 (enclosed by green box) correspond to the reconstruction of original images.

In Figure 2, Figure 3 and Figure 4, we provide results of image editing on face images from five subjects as examples. The image editing manifested here is based on the clustering and representation learning results of VaDA. Given the space limitations, we have placed the image editing results based on VaDE and VAE+GMM in the Supplementary Materials (Section S2). To make consistent comparison with the other methods, the clustering rule VaDA-X is applied.

Figure 2 corresponds to the results when the subjects are separated into two clusters. We can see smooth transition between dark and light backgrounds from cluster k = 0 to k = 1. It is also noticeable that little by little the hair becomes shorter while other facial attributes are largely retained.

Figure 3 manifests the image editing results when three clusters are assumed. Again, the dominant variations are from the background and the hair. From cluster k = 0 to k = 1, the background becomes lighter, the hair gets shorter and the hair color is slightly deepened. From cluster k  =  1 to k = 2, reversed changes are presented. Nevertheless, the left side of image editing from k = 0 to k = 1 shows a warmer color palette than the right side of image editing from k = 1 to k = 2. Interestingly, this subtle difference can be validated by image editing from k = 2 to k = 0, where the overall color tone shifts towards warmer hues. In addition, there also exhibits slight variation on the fringe. While the fringe gets thinner from k = 1 to k = 2, it becomes heavier from k = 2 to k = 0. This therefore corresponds to the little change of fringe from cluster k = 0 to k = 1.

**Figure 5:**
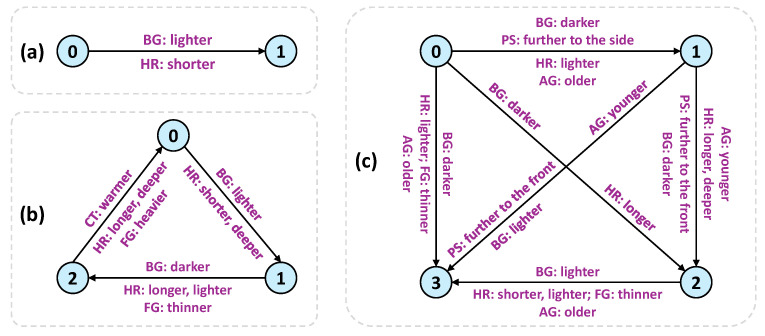
Dominant visual transitions directed by the concept vectors from VaDA when K = 2 (**a**), K = 3 (**b**) and K = 4 (**c**). The blue nodes represent the clusters. We use BG, CT, PS, HR, FG and AG as an abbreviation for background, color tone, face pose, hair, fringe and age, respectively.

Figure 4 shows the image editing results decoded by the six concept vectors from a four-cluster separation of the subjects. Besides the background changes and the hair length and color transitions, we detect two additional meaningful variation axes: age and face pose. It can be seen clearly that there are transitions on age from cluster k = 0 to k = 1, from k = 0 to k = 3, from k = 2 to k = 1 and from k = 2 to k = 3 with the appearance getting older. The pattern of transition from cluster k = 0 to k = 1 shares similarity with that from k = 0 to k = 3, where the background becomes darker along with the growing age. Nevertheless, difference exists between cluster k = 1 and k = 3. The appearance gets old little by little from k = 3 to k = 1, which completes a circle from k = 0 to k = 3, from k = 3 to k = 1 and finally from k = 1 to k = 0 with the age getting older and older and then returning young. The similar circle can be figured out from cluster k = 2 to k = 3, from k = 3 to k = 1 and finally from k = 1 to k = 2 but with a reversed transition on the darkness of background. Additionally, smooth transitions on the face pose can be found from cluster k = 1 to k = 0, from k = 1 to k = 2 and from k = 1 to k = 3, where the faces gradually turn from profile to full front. This detection illustrates that the face images from cluster k = 1 could be characterized as facing sideways. We summarize the transitions between different clusters in Figure 5.

Compared to the proposed method, the clustering performances of VaDE and VAE+GMM are largely unsatisfactory. The VaDE method which performs representation learning and clustering simultaneously gives distinguishable clusters under different settings of the number of clusters. However, it has limited generalization capability as shown in case K = 3 (Figure S2) and K = 4 (Figure S3). The approach to cluster k = 0 produces images that gradually blur. Generally, the latent space learned by VAE is not responsive to the clustering result of GMM (Figures S4–S6). There is nearly imperceptible variation when changing the α value.

### 5.3. Image Reconstruction and Generation

**Figure 6:**
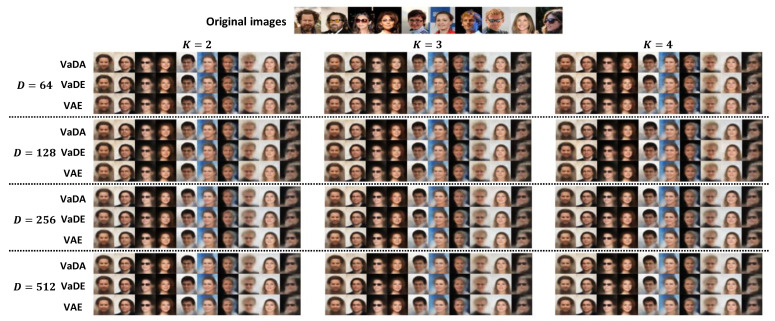
Image reconstruction by method VaDA, VaDE and VAE under different settings of *D* and *K*.

The image reconstruction results are compared between the methods VaDA, VaDE and VAE. We map the testing images to the latent space using the X-encoder and decode them back to the image space using the X-decoder. Ten reconstruction examples are presented in Figure 6. The three methods sharing the same encoder and decoder structures exhibit comparable performance and there is nearly no difference that can be identified given the latent dimensions *D* and the number of clusters *K*. While varying the setting of *K* has little impact on the image reconstruction, increasing the latent dimensions *D* gradually smooths the images. The overall results demonstrate the effectiveness of the X-encoder and X-decoder established in our experiments, which can capture the main properties of the image data.

**Figure 7:**
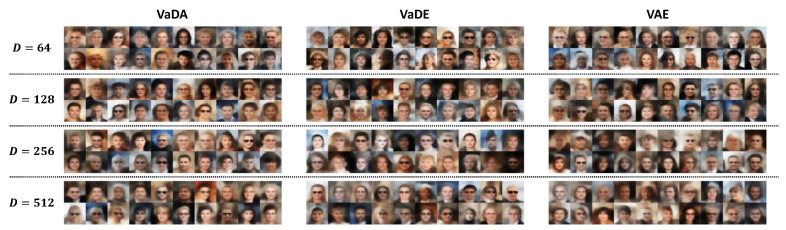
Image generation by method VaDA, VaDE and VAE under different settings of latent dimensions *D* given K = 2.

Figure 7 shows the face images generated by VaDA, VaDE and VAE with K = 2. Random face images were obtained by decoding the latent vectors drawn from the estimated latent space. Overall, compared with VaDA, the methods VaDE and VAE are less advantaged in out-of-sample generalization. Even though the generated images by VaDA are not perfect and tend to be blurry on the hair, they are more realistic on the face color and have compatible and harmonious overall appearance than the images generated by VaDE and VAE. VaDE is more likely to produce weird faces in our experiments and the images generated by VAE tend to be blurrier though the overall spatial face structures are preserved. Nevertheless, the three methods show improved data generating performance as the latent dimensions *D* is increased. The out-of-sample generalization performance relies heavily on correct modeling of the latent space structures. The outperforming of VaDA is in line with expectation as it has a better-structured latent space allowing within-subject dependence and unifying the latent information from outcome and features.

## 6. Conclusions

In this paper, we developed a generative auto-encoding approach to longitudinal data analysis. VaDA retains the strengths of VAE to learn well-structured latent space and is an enhanced version of VaDE to accommodate the nature of longitudinal data. Moreover, it goes beyond the GEEs based marginal modeling approach by forging a deep “alliance” across measurements to incorporate more complicated dependence structures. VaDA integrates prediction, clustering and representation learning in a unified AEVB framework, which alleviates the loss of information between different stages and fortifies interaction and coordination across all parts of the model.

Synthetic and real-world data experiments were implemented. The results demonstrated the robustness and effectiveness of the proposed method, which achieved high prediction accuracy and showed excellent ability in recovering the underlying grouping of the data under varied assumptions of data generating mechanism. The VaDA approach well captured the latent structure of CelebA longitudinal data, where we obtained meaningful clustering results of the face images. Moreover, it exhibited high generalization capability. The generated face images have more realistic and harmonious appearance than those from VAE or VaDE.

Nevertheless, there is still room further improvements. In the experiments, we have used given decoder and encoder network structures. For synthetic data, the networks similar to the ones in the data generating process were adopted. For CelebA data, we used the available convolution neural network structures established in previous studies for face images and a simple dense network to model the relationship between outcomes. A pilot study was conducted to investigate the impact of redundancy in encoder and decoder network architectures (see Section S3 in Supplementary Materials). While the redundancy was found to degrade the performance of VaDA, incorporating “dropout” layers [38] could mitigate this issue, resulting in a calibrated model. These findings highlight the need for a more comprehensive exploration of network structures in order to optimize the performance of VaDA for real-world applications.

Another potential enhancement of the method is to weight the different variational loss terms like the practice in [39], especially to consider the balance between reconstruction loss terms on response and covariates. In addition, a thorough analysis of the identifiability of VaDA, while beyond the scope of this work, remains an important theoretical question for further research. The study in [40] has demonstrated the identifiability properties of deep generative models like VAE and VaDE up to an affine transformation under moderate conditions. Our future work should explore the potentials of extending these results to VaDA to strengthen its theoretical foundation.

In current work, we have not considered the handling of missing data on covariates or response, which frequently arise in longitudinal study. Improper treatment of missingness could introduce bias under certain missing data mechanisms [41]. Nevertheless, the generative modeling framework of VaDA can be extended immediately to the missing data cases, where missing data mechanism can be integrated naturally into the ELBO to derive a systematic treatment. Such enhancement will strengthen the robustness and generalizability of current model.

While the proposed VaDA approach processes high flexibility in handling the high-dimensional longitudinal data, there are still limitations that it is only suitable to datasets with equal-length measurements across subjects (these datasets often appear in well scheduled and organized longitudinal experiments). Nevertheless, the inference framework of VaDA provides a solid foundation for combining the efforts of VAE with alternative classical methods for other types of longitudinal data. A potential is to go beyond the generalized neural network mixed effects model in [23] by extending it to a full generative framework, which is a part of our ongoing work.

The present study introduces a novel longitudinal perspective to the widely recognized CelebA dataset by reorganizing its structure and applying the VaDA method for improved representation learning, clustering and image generation. This perspective could offer a meaningful contribution to the pattern discovery based on CelebA and other benchmark vision datasets. Future work could explore the applicability of the method to longitudinal data with more complex and naturally evolving spatio-temporal dynamics such as those in clinical or behavioral studies. This may also involve extending the proposed framework to handle challenges including missing data and irregular length of measurements as noted in our earlier discussion, therefore further enhancing the VaDA’s practical utility in more real-world longitudinal studies.

## Figures and Tables

**Table 1:** Prediction results on the testing datasets evaluated by the mean square error with varied settings of the latent dimensions *D* when the number of clusters K = 3.

Scenario	Method	*D* = 2	*D* = 5	*D* = 10	*D* = 20	*D* = 100
11	VaDA *	0.281 ± 0.117	0.122 ± 0.045	0.068 ± 0.007	0.059 ± 0.005	0.062 ± 0.008
	VaDE+MM0	0.769 ± 0.026	0.749 ± 0.025	0.730 ± 0.021	0.728 ± 0.020	0.740 ± 0.022
	VaDE+MM1	0.773 ± 0.028	0.752 ± 0.027	0.732 ± 0.022	0.729 ± 0.020	0.735 ± 0.021
	VaDE+MM2	0.774 ± 0.027	0.751 ± 0.026	0.729 ± 0.020	0.726 ± 0.021	0.726 ± 0.021
	VAE+GMM+MM0	0.725 ± 0.020	0.726 ± 0.021	0.726 ± 0.020	0.728 ± 0.021	0.740 ± 0.022
	VAE+GMM+MM1	0.728 ± 0.020	0.729 ± 0.021	0.729 ± 0.020	0.730 ± 0.021	0.734 ± 0.021
	VAE+GMM+MM2	0.725 ± 0.020	0.726 ± 0.021	0.725 ± 0.021	0.726 ± 0.021	0.726 ± 0.021
	GMM+MM0	—	—	—	—	0.787 ± 0.078
	GMM+MM1	—	—	—	—	0.757 ± 0.044
	GMM+MM2	—	—	—	—	0.726 ± 0.021
01	VaDA	0.233 ± 0.097	0.069 ± 0.040	0.053 ± 0.009	0.048 ± 0.011	0.048 ± 0.010
	VaDE+MM0	0.397 ± 0.062	0.274 ± 0.066	0.092 ± 0.057	0.007 ± 0.004	0.005 ± 0.001
	VaDE+MM1	0.781 ± 0.137	0.470 ± 0.192	0.119 ± 0.098	0.007 ± 0.004	0.005 ± 0.001
	VaDE+MM2	0.782 ± 0.139	0.541 ± 0.208	0.120 ± 0.097	0.007 ± 0.004	0.005 ± 0.001
	VAE+GMM+MM0	0.046 ± 0.001	0.035 ± 0.001	0.015 ± 0.024	0.013 ± 0.029	0.015 ± 0.025
	VAE+GMM+MM1	0.046 ± 0.001	0.035 ± 0.001	0.015 ± 0.024	0.013 ± 0.029	0.015 ± 0.025
	VAE+GMM+MM2	0.046 ± 0.001	0.035 ± 0.001	0.015 ± 0.024	0.013 ± 0.029	0.015 ± 0.025
	GMM+MM0	—	—	—	—	0.012 ± 0.019
	GMM+MM1	—	—	—	—	0.012 ± 0.019
	GMM+MM2	—	—	—	—	0.012 ± 0.019
10	VaDA	0.092 ± 0.005	0.092 ± 0.005	0.092 ± 0.004	0.092 ± 0.004	0.091 ± 0.005
	VaDE+MM0	1.003 ± 0.026	1.003 ± 0.026	1.004 ± 0.026	73.75 ± 243.5	3002 ± 2948
	VaDE+MM1	1.004 ± 0.026	1.004 ± 0.026	1.005 ± 0.026	77.57 ± 262.2	3023 ± 2961
	VaDE+MM2	1.002 ± 0.026	1.003 ± 0.026	1.002 ± 0.026	15.03 ± 23.92	1108 ± 2936
	VAE+GMM+MM0	1.003 ± 0.026	1.003 ± 0.026	1.005 ± 0.026	88.12 ± 290.5	3961 ± 1976
	VAE+GMM+MM1	1.004 ± 0.026	1.005 ± 0.026	1.006 ± 0.026	89.30 ± 293.8	4029 ± 2010
	VAE+GMM+MM2	1.002 ± 0.026	1.003 ± 0.026	1.003 ± 0.026	19.52 ± 56.10	933.7 ± 419.5
	GMM+MM0	—	—	—	—	1.020 ± 0.028
	GMM+MM1	—	—	—	—	1.022 ± 0.028
	GMM+MM2	—	—	—	—	1.004 ± 0.026
00	VaDA	0.108 ± 0.007	0.109 ± 0.011	0.108 ± 0.010	0.106 ± 0.008	0.103 ± 0.003
	VaDE+MM0	0.112 ± 0.017	0.108 ± 0.013	0.107 ± 0.016	5.987 ± 16.04	169.1 ± 79.02
	VaDE+MM1	0.112 ± 0.017	0.111 ± 0.029	0.107 ± 0.016	5.993 ± 16.12	168.4 ± 79.18
	VaDE+MM2	0.112 ± 0.017	0.111 ± 0.024	0.107 ± 0.016	5.959 ± 15.93	168.2 ± 78.26
	VAE+GMM+MM0	0.104 ± 0.011	0.103 ± 0.011	0.101 ± 0.012	7.666 ± 24.27	385.4 ± 88.45
	VAE+GMM+MM1	0.104 ± 0.011	0.103 ± 0.011	0.101 ± 0.012	7.555 ± 24.18	386.4 ± 91.34
	VAE+GMM+MM2	0.104 ± 0.011	0.103 ± 0.011	0.101 ± 0.012	7.558 ± 24.06	385.2 ± 89.04
	GMM+MM0	—	—	—	—	0.018 ± 0.026
	GMM+MM1	—	—	—	—	0.018 ± 0.026
	GMM+MM2	—	—	—	—	0.018 ± 0.026

* Visual highlighting with gray background is applied to the results of our proposed method to facilitate quick identification and comparison with baseline methods.

**Table 2:** Prediction results on the testing datasets evaluated by the mean square error with varied settings of the number of clusters *K* when the latent dimensions D=10.

Scenario	Method	*K* = 2	*K* = 3	*K* = 4
11	VaDA *	0.071 ± 0.020	0.068 ± 0.007	0.067 ± 0.007
	VaDE+MM0	0.731 ± 0.022	0.730 ± 0.021	0.734 ± 0.021
	VaDE+MM1	0.734 ± 0.024	0.732 ± 0.022	0.737 ± 0.022
	VaDE+MM2	0.730 ± 0.022	0.729 ± 0.020	0.734 ± 0.021
	VAE+GMM+MM0	0.732 ± 0.022	0.726 ± 0.020	0.727 ± 0.020
	VAE+GMM+MM1	0.735 ± 0.024	0.729 ± 0.020	0.729 ± 0.020
	VAE+GMM+MM2	0.731 ± 0.022	0.725 ± 0.021	0.725 ± 0.021
	GMM+MM0	0.753 ± 0.033	0.787 ± 0.078	0.838 ± 0.115
	GMM+MM1	0.742 ± 0.024	0.757 ± 0.044	0.782 ± 0.061
	GMM+MM2	0.726 ± 0.021	0.726 ± 0.021	0.727 ± 0.020
01	VaDA	0.051 ± 0.007	0.053 ± 0.009	0.051 ± 0.006
	VaDE+MM0	0.114 ± 0.031	0.092 ± 0.057	0.129 ± 0.046
	VaDE+MM1	0.118 ± 0.042	0.119 ± 0.098	0.154 ± 0.085
	VaDE+MM2	0.120 ± 0.042	0.120 ± 0.097	0.162 ± 0.097
	VAE+GMM+MM0	0.120 ± 0.037	0.015 ± 0.024	0.007 ± 0.001
	VAE+GMM+MM1	0.120 ± 0.037	0.015 ± 0.024	0.007 ± 0.001
	VAE+GMM+MM2	0.120 ± 0.037	0.015 ± 0.024	0.007 ± 0.001
	GMM+MM0	0.028 ± 0.025	0.012 ± 0.019	0.007 ± 0.008
	GMM+MM1	0.028 ± 0.025	0.012 ± 0.019	0.007 ± 0.008
	GMM+MM2	0.028 ± 0.025	0.012 ± 0.019	0.007 ± 0.008
10	VaDA	0.096 ± 0.012	0.092 ± 0.004	0.093 ± 0.008
	VaDE+MM0	1.004 ± 0.026	1.004 ± 0.026	1.003 ± 0.026
	VaDE+MM1	1.005 ± 0.026	1.005 ± 0.026	1.005 ± 0.026
	VaDE+MM2	1.002 ± 0.026	1.002 ± 0.026	1.003 ± 0.026
	VAE+GMM+MM0	1.004 ± 0.026	1.005 ± 0.026	1.005 ± 0.027
	VAE+GMM+MM1	1.006 ± 0.026	1.006 ± 0.026	1.006 ± 0.027
	VAE+GMM+MM2	1.003 ± 0.026	1.003 ± 0.026	1.003 ± 0.026
	GMM+MM0	1.015 ± 0.026	1.020 ± 0.028	1.020 ± 0.028
	GMM+MM1	1.016 ± 0.026	1.022 ± 0.028	1.021 ± 0.028
	GMM+MM2	1.003 ± 0.026	1.004 ± 0.026	1.004 ± 0.026
00	VaDA	0.107 ± 0.006	0.108 ± 0.010	0.110 ± 0.015
	VaDE+MM0	0.105 ± 0.011	0.107 ± 0.016	0.104 ± 0.010
	VaDE+MM1	0.105 ± 0.011	0.107 ± 0.016	0.104 ± 0.010
	VaDE+MM2	0.105 ± 0.011	0.107 ± 0.016	0.104 ± 0.010
	VAE+GMM+MM0	0.104 ± 0.011	0.101 ± 0.012	0.097 ± 0.004
	VAE+GMM+MM1	0.104 ± 0.011	0.101 ± 0.012	0.097 ± 0.004
	VAE+GMM+MM2	0.104 ± 0.011	0.101 ± 0.012	0.097 ± 0.004
	GMM+MM0	0.069 ± 0.027	0.018 ± 0.026	0.022 ± 0.027
	GMM+MM1	0.069 ± 0.027	0.018 ± 0.026	0.022 ± 0.027
	GMM+MM2	0.069 ± 0.027	0.018 ± 0.026	0.022 ± 0.027

* Visual highlighting with gray background is applied to the results of our proposed method to facilitate quick identification and comparison with baseline methods.

**Table 3:** Clustering results on the testing datasets evaluated by the adjusted Rand index with varied settings of the latent dimensions *D* when the number of clusters K=3.

Scenario	Method	*D* = 2	*D* = 5	*D* = 10	*D* = 20	*D* = 100
11	VaDA-YX *	0.986 ± 0.073	0.942 ± 0.147	0.957 ± 0.129	0.973 ± 0.102	0.914 ± 0.172
	VaDA-X	0.986 ± 0.073	0.942 ± 0.147	0.957 ± 0.129	0.973 ± 0.102	0.914 ± 0.172
	VaDE	0.381 ± 0.144	0.420 ± 0.155	0.782 ± 0.217	0.985 ± 0.079	1.000 ± 0.000
	VAE+GMM	1.000 ± 0.000	0.982 ± 0.100	1.000 ± 0.000	0.946 ± 0.165	0.930 ± 0.146
	GMM	—	—	—	—	0.816 ± 0.265
01	VaDA-YX	1.000 ± 0.000	1.000 ± 0.000	0.972 ± 0.105	0.987 ± 0.071	0.913 ± 0.174
	VaDA-X	1.000 ± 0.000	1.000 ± 0.000	0.972 ± 0.105	0.987 ± 0.071	0.913 ± 0.174
	VaDE	0.309 ± 0.159	0.412 ± 0.136	0.731 ± 0.201	1.000 ± 0.001	1.000 ± 0.000
	VAE+GMM	1.000 ± 0.000	1.000 ± 0.000	0.945 ± 0.168	0.964 ± 0.138	0.921 ± 0.169
	GMM	—	—	—	—	0.816 ± 0.265
10	VaDA-YX	0.942 ± 0.147	0.956 ± 0.132	0.957 ± 0.131	0.956 ± 0.133	0.972 ± 0.104
	VaDA-X	0.942 ± 0.147	0.956 ± 0.132	0.957 ± 0.131	0.956 ± 0.133	0.972 ± 0.104
	VaDE	0.611 ± 0.273	0.540 ± 0.247	0.590 ± 0.170	0.592 ± 0.292	0.672 ± 0.271
	VAE+GMM	0.802 ± 0.265	0.853 ± 0.248	0.816 ± 0.265	0.908 ± 0.209	0.852 ± 0.249
	GMM	—	—	—	—	0.914 ± 0.176
00	VaDA-YX	0.930 ± 0.157	0.940 ± 0.147	1.000 ± 0.000	0.986 ± 0.077	0.942 ± 0.148
	VaDA-X	0.930 ± 0.157	0.942 ± 0.148	1.000 ± 0.000	0.986 ± 0.077	0.942 ± 0.148
	VaDE	0.655 ± 0.216	0.573 ± 0.253	0.631 ± 0.212	0.651 ± 0.218	0.580 ± 0.207
	VAE+GMM	0.778 ± 0.260	0.816 ± 0.264	0.778 ± 0.276	0.743 ± 0.280	0.926 ± 0.192
	GMM	—	—	—	—	0.912 ± 0.179

* Visual highlighting with gray background is applied to the results of our proposed method to facilitate quick identification and comparison with baseline methods.

**Table 4:** Clustering results on the testing datasets evaluated by the adjusted Rand index with varied settings of the number of clusters *K* when the latent dimensions D=10.

Scenario	Method	*K* = 2	*K* = 3	*K* = 4
11	VaDA-YX *	0.569 ± 0.023	0.957 ± 0.129	0.984 ± 0.084
	VaDA-X	0.569 ± 0.023	0.957 ± 0.129	0.984 ± 0.084
	VaDE	0.533 ± 0.058	0.782 ± 0.217	0.455 ± 0.141
	VAE+GMM	0.567 ± 0.022	1.000 ± 0.000	0.871 ± 0.081
	GMM	0.567 ± 0.025	0.816 ± 0.265	0.875 ± 0.178
01	VaDA-YX	0.574 ± 0.027	0.972 ± 0.105	0.986 ± 0.073
	VaDA-X	0.574 ± 0.027	0.972 ± 0.105	0.986 ± 0.073
	VaDE	0.564 ± 0.033	0.731 ± 0.201	0.561 ± 0.151
	VAE+GMM	0.577 ± 0.025	0.945 ± 0.168	0.890 ± 0.088
	GMM	0.567 ± 0.025	0.816 ± 0.265	0.875 ± 0.178
10	VaDA-YX	0.569 ± 0.015	0.957 ± 0.131	1.000 ± 0.000
	VaDA-X	0.569 ± 0.015	0.957 ± 0.131	1.000 ± 0.000
	VaDE	0.570 ± 0.024	0.590 ± 0.170	0.616 ± 0.232
	VAE+GMM	0.572 ± 0.019	0.816 ± 0.265	0.823 ± 0.173
	GMM	0.569 ± 0.019	0.914 ± 0.176	0.877 ± 0.200
00	VaDA-YX	0.572 ± 0.021	1.000 ± 0.000	0.957 ± 0.128
	VaDA-X	0.572 ± 0.021	1.000 ± 0.000	0.957 ± 0.128
	VaDE	0.577 ± 0.023	0.631 ± 0.212	0.636 ± 0.147
	VAE+GMM	0.571 ± 0.023	0.778 ± 0.276	0.820 ± 0.181
	GMM	0.568 ± 0.019	0.912 ± 0.179	0.873 ± 0.198

* Visual highlighting with gray background is applied to the results of our proposed method to facilitate quick identification and comparison with baseline methods.

**Table 5:** Prediction results on the CelebA training/testing dataset evaluated by the classification error rate with varied settings of the latent dimensions *D* and the number of clusters *K*.

Dimensions	Method	*K* = 2	*K* = 3	*K* = 4
D=64	VaDA *	0.102/0.070	0.098/0.067	0.097/0.071
	VaDE+MM0	0.155/0.180	0.146/0.168	0.143/0.191
	VaDE+MM1	0.172/0.177	0.162/0.180	0.160/0.212
	VAE+GMM+MM0	0.149/0.189	0.153/0.185	0.149/0.187
	VAE+GMM+MM1	0.482/0.479	0.364/0.406	0.167/0.210
D=128	VaDA	0.097/0.077	0.108/0.082	0.095/0.060
	VaDE+MM0	0.129/0.175	0.134/0.188	0.106/0.210
	VaDE+MM1	0.155/0.184	0.165/0.208	0.161/0.239
	VAE+GMM+MM0	0.131/0.174	0.122/0.194	0.114/0.206
	VAE+GMM+MM1	0.195/0.233	0.129/0.187	0.351/0.397
D=256	VaDA	0.090/0.069	0.090/0.066	0.098/0.069
	VaDE+MM0	0.102/0.208	0.094/0.206	0.090/0.228
	VaDE+MM1	0.128/0.231	0.124/0.213	0.092/0.229
	VAE+GMM+MM0	0.097/0.204	0.063/0.268	0.040/0.313
	VAE+GMM+MM1	0.129/0.234	0.070/0.271	0.039/0.313
D=512	VaDA	0.094/0.070	0.103/0.077	0.098/0.080
	VaDE+MM0	0.045/0.350	0.000/0.361	0.048/0.337
	VaDE+MM1	0.045/0.359	0.000/0.370	0.068/0.352
	VAE+GMM+MM0	0.051/0.324	0.000/0.372	0.000/0.419
	VAE+GMM+MM1	0.051/0.324	0.000/0.377	0.000/0.421

* Visual highlighting with gray background is applied to the results of our proposed method to facilitate quick identification and comparison with baseline methods.

**Figure A1:**
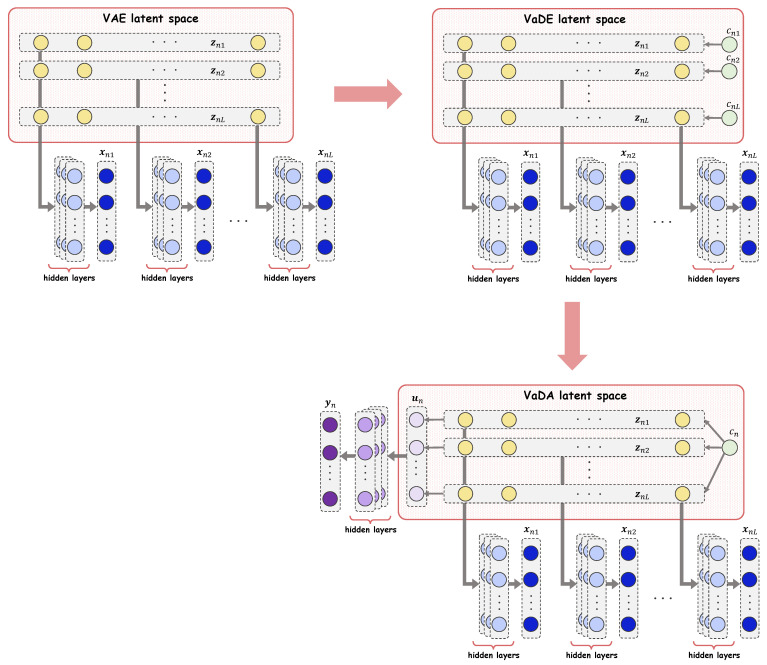
The evolution of model structures between the standard VAE, VaDE and VaDA.

**Figure A2:**
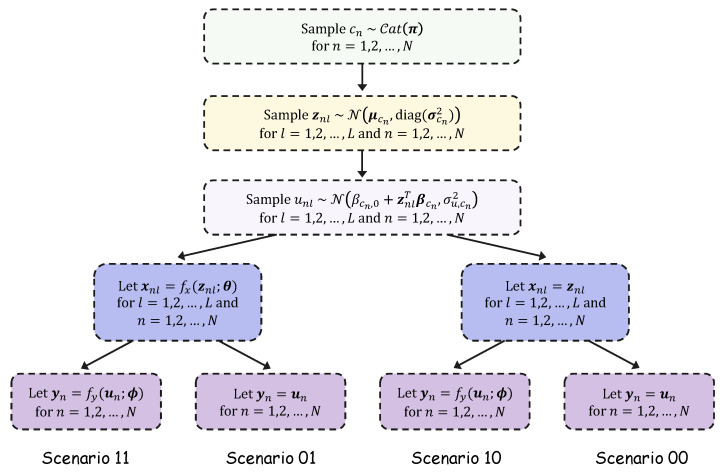
Data generating procedures for the synthetic datasets in Scenarios 11, 01, 10 and 00.

**Figure A3:**
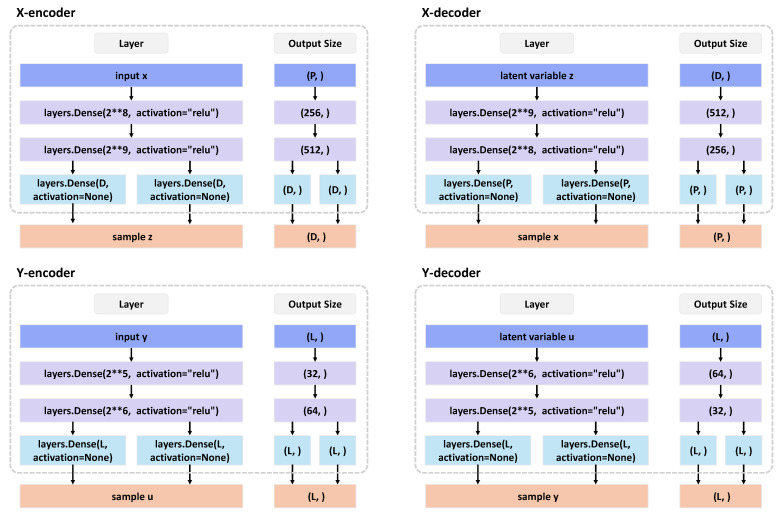
Architectures of encoder and decoder networks used in the experiments for synthetic data. The network layers are represented by their corresponding Python code.

**Figure A4:**
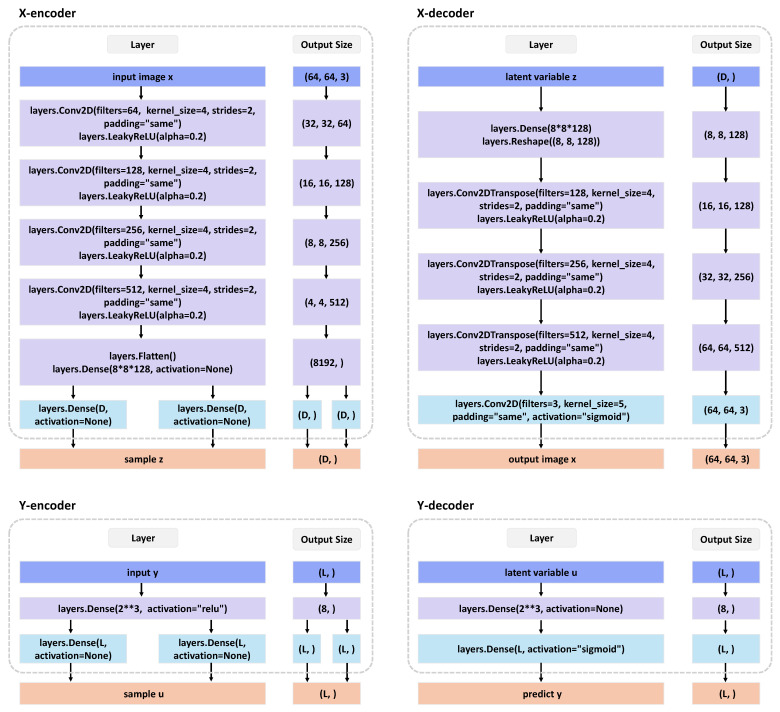
Architectures of encoder and decoder networks used in the experiments for CelebA data. The network layers are represented by their corresponding Python code.

**Figure A5:**
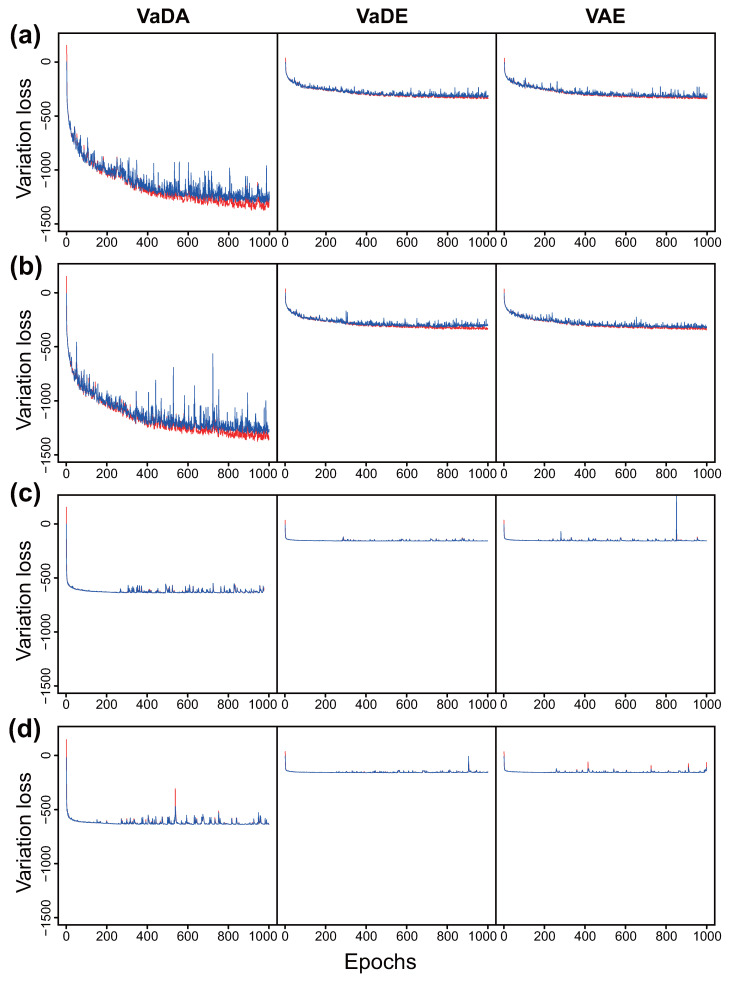
Trends of training loss (red line) and validation loss (blue line) in the synthetic scenarios 11 (**a**), 01 (**b**), 10 (**c**), and 00 (**d**).

**Figure A6:**
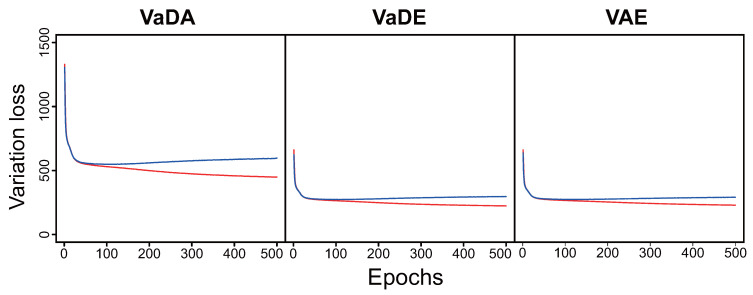
Trends of training loss (red line) and validation loss (blue line) for the CelebA longitudinal dataset.

**Figure A7:**
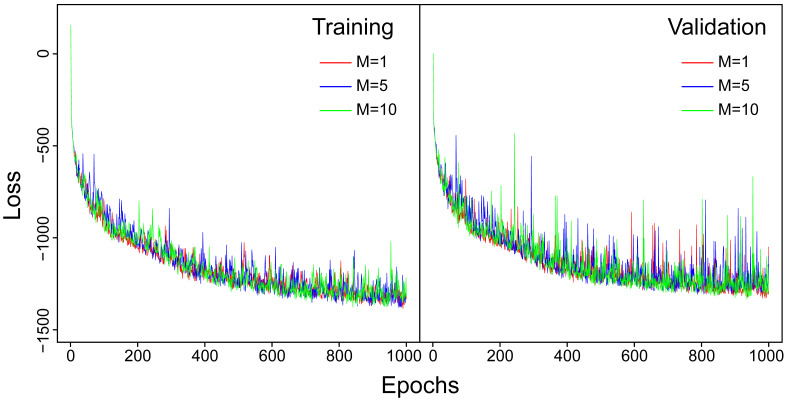
Comparison of the training loss and validation loss of VaDA with different MC sample size *M* under the setting of K=3 and D=10.

## Data Availability

The original CelebFaces Attributes (CelebA) Dataset was downloaded from the webpage: https://mmlab.ie.cuhk.edu.hk/projects/CelebA.html (accessed on 12 July 2025). The deep learning methods VaDA, VaDE and VAE were implemented based on the programming language Python (version: 3.12.4) with the backbone “TensorFlow”. The marginal model, the GMM-based clustering and the PCA were implemented based on the programming language R (version: 4.5.1) using the packages “geepack” and “elasticnet”. The codes for the experimental study are available upon request from the corresponding authors.

## Supplementary Materials

The following supporting information can be downloaded at https://www.mdpi.com/article/10.3390/e28010113/s1. Figure S1: image editing by concept vector between each pair of clusters based on the results of VaDE with K = 2; Figure S2: image editing by concept vector between each pair of clusters based on the results of VaDE with K = 3; Figure S3: image editing by concept vector between each pair of clusters based on the results of VaDE with K = 4; Figure S4: image editing by concept vector between each pair of clusters based on the results of VAE+GMM with K = 2; Figure S5: image editing by concept vector between each pair of clusters based on the results of VAE+GMM with K = 3; Figure S6: image editing by concept vector between each pair of clusters based on the results of VAE+GMM with K = 4; Figure S7: architectures of encoder and decoder networks in VaDA for synthetic data with redundancy; Figure S8: architectures of encoder and decoder networks in VaDA for synthetic data with dropout; Table S1: prediction results on the training datasets evaluated by the mean square error with varied settings of the latent dimensions *D* when the number of clusters K = 3; Table S2: prediction results on the training datasets evaluated by the mean square error with varied settings of the number of clusters *K* when the latent dimensions D = 10; Table S3: clustering results on the training datasets evaluated by the adjusted Rand index with varied settings of the latent dimensions *D* when the number of clusters K = 3; Table S4: clustering results on the training datasets evaluated by the adjusted Rand index with varied settings of the number of clusters *K* when the latent dimensions D = 10; Table S5: the performance of VaDA with normal, redundant and dropout included network structures evaluated on the metrics of adjusted Rand index for clustering results from VaDA-YX (ARI-YX) and from VaDA-X (ARI-X), mean square error for prediction accuracy (MSE).

## Appendix A. Derive Terms in KL Divergence

In this section, we explain how the final form of $D_{\mathrm{KL}}\left[q\left(u_{n},Z_{n},c_{n}|y_{n},X_{n}\right)\left|\right|p\left(u_{n},Z_{n},c_{n}\right)\right]$ is derived. Following the definition of KL divergence, we have

(A1) $$\begin{array}{cc} D_{\mathrm{KL}}[ & q\left(u_{n},Z_{n},c_{n}|y_{n},X_{n}\right)\left|\right|p\left(u_{n},Z_{n},c_{n}\right)]\\ \; & =-E\left[\log p(u_{n}|Z_{n},c_{n})\right]-E\left[\log p(Z_{n}|c_{n})\right]-E\left[\log p(c_{n})\right]\\ \; & \;+E\left[\log q(u_{n}|y_{n})\right]+E\left[\log q(Z_{n}|X_{n})\right]+E\left[\log q(c_{n}|y_{n},X_{n})\right], \end{array}$$

where the expectation is taken w.r.t. $q(u_{n},Z_{n},c_{n}|y_{n},X_{n})$ with assumed decomposition given in (2). Computation of the terms in (A1) is given as follows.

(1) $E\left[\log p(u_{n}|Z_{n},c_{n})\right]$

(A2) $$\begin{array}{cc} E\left[\log p(u_{n}|Z_{n},c_{n})\right] & =\sum_{k=1}^{K}{\tilde{\pi }}_{k}E\left[\log p(u_{n}|Z_{n},c_{n}=k)\right]\\ \; & =\sum_{k=1}^{K}{\tilde{\pi }}_{k}\sum_{l=1}^{L}E\left[\log p(u_{nl}|z_{nl},c_{n}=k)\right]\\ \; & =\sum_{k=1}^{K}{\tilde{\pi }}_{k}\sum_{l=1}^{L}E\left[\log N\left(u_{nl};\beta_{k,0}+z_{nl}^{T}\beta_{k},\sigma_{u,k}^{2}\right)\right]\\ \; & =\sum_{k=1}^{K}{\tilde{\pi }}_{k}\sum_{l=1}^{L}E\left[-\frac{1}{2}\log\sigma_{u,k}^{2}-\frac{1}{2\sigma_{u,k}^{2}}{\left(u_{nl}-\beta_{k,0}-z_{nl}^{T}\beta_{k}\right)}^{2}\right]+const. \end{array}$$

As $q(u_{nl}|y_{n})$ and $q(z_{nl}|x_{nl})$ are both Gaussian distribution, i.e.,

$$\begin{array}{cc} \; & q\left(u_{nl}|y_{n}\right)=N\left(u_{nl};{\tilde{\mu }}_{u,l},{\tilde{\sigma }}_{u,l}^{2}\right),\\ \; & q\left(z_{nl}|x_{nl}\right)=N\left(z_{nl};{\tilde{\mu }}_{z},\mathrm{diag}\left({\tilde{\sigma }}_{z}^{2}\right)\right), \end{array}$$

we have

$$\begin{array}{cc} \; & E\left(u_{nl}\right)={\tilde{\mu }}_{u,l},\;E\left(u_{nl}^{2}\right)={\tilde{\mu }}_{u,l}^{2}+{\tilde{\sigma }}_{u,l}^{2},\\ \; & E\left(z_{nl}\right)={\tilde{\mu }}_{z},\;E\left(z_{nl}z_{nl}^{T}\right)={\tilde{\mu }}_{z}{\tilde{\mu }}_{z}^{T}+\mathrm{diag}\left({\tilde{\sigma }}_{z}^{2}\right). \end{array}$$

Using these results to compute (A2), it follows

$$E\left[\log p(u_{n}|Z_{n},c_{n})\right]=\sum_{k=1}^{K}{\tilde{\pi }}_{k}\sum_{l=1}^{L}\left\{-\frac{1}{2}\log\sigma_{u,k}^{2}-\frac{1}{2\sigma_{u,k}^{2}}\left[{\left({\tilde{\mu }}_{u,l}-\beta_{k,0}-{\tilde{\mu }}_{z}^{T}\beta_{k}\right)}^{2}+{\tilde{\sigma }}_{u,l}^{2}+\beta_{k}^{T}\mathrm{diag}\left({\tilde{\sigma }}_{z}^{2}\right)\beta_{k}\right]\right\}+const.$$

(2) $E\left[\log p(Z_{n}|c_{n})\right]$

$$\begin{array}{cc} E\left[\log p(Z_{n}|c_{n})\right] & =\sum_{k=1}^{K}{\tilde{\pi }}_{k}E\left[\log p(Z_{n}|c_{n}=k)\right]\\ \; & =\sum_{k=1}^{K}{\tilde{\pi }}_{k}\sum_{l=1}^{L}E\left[\log p(z_{nl}|c_{n}=k)\right]\\ \; & =\sum_{k=1}^{K}{\tilde{\pi }}_{k}\sum_{l=1}^{L}\sum_{d=1}^{D}E\left[\log N\left(z_{nld};\mu_{k,d},\sigma_{k,d}^{2}\right)\right]\\ \; & =\sum_{k=1}^{K}{\tilde{\pi }}_{k}\sum_{l=1}^{L}\sum_{d=1}^{D}E\left[-\frac{1}{2}\log\sigma_{k,d}^{2}-\frac{1}{2\sigma_{k,d}^{2}}{\left(z_{nld}-\mu_{k,d}\right)}^{2}\right]+const. \end{array}$$

Using $E\left(z_{nld}\right)={\tilde{\mu }}_{z,d}$ and $E\left(z_{nld}^{2}\right)={\tilde{\mu }}_{z,d}^{2}+{\tilde{\sigma }}_{z,d}^{2}$, we have

$$E\left[\log p(Z_{n}|c_{n})\right]=\sum_{k=1}^{K}{\tilde{\pi }}_{k}\sum_{l=1}^{L}\sum_{d=1}^{D}\left\{-\frac{1}{2}\log\sigma_{k,d}^{2}-\frac{1}{2\sigma_{k,d}^{2}}\left[{\left({\tilde{\mu }}_{z,d}-\mu_{k,d}\right)}^{2}+{\tilde{\sigma }}_{z,d}^{2}\right]\right\}+const.$$

(3) $E\left[\log q(u_{n}|y_{n})\right]$

Using Lemma 1 of Appendix B in [26], we can directly obtain

$$E\left[\log q(u_{n}|y_{n})\right]=-\frac{1}{2}\sum_{l=1}^{L}\log{\tilde{\sigma }}_{u,l}^{2}+const.$$

(4) $E\left[\log q(Z_{n}|X_{n})\right]$

Using again the Lemma 1 of Appendix B in [26], we obtain

$$E\left[\log q(Z_{n}|X_{n})\right]=\sum_{l=1}^{L}E\left[\log q(z_{nl}|x_{nl})\right]=-\frac{1}{2}\sum_{l=1}^{L}\sum_{d=1}^{D}\log{\tilde{\sigma }}_{z,d}^{2}+const.$$

(5) $E\left[\log p(c_{n})\right]$ and $E\left[\log q(c_{n}|y_{n},X_{n})\right]$

$$\begin{array}{cc} \; & E\left[\log p(c_{n})\right]=\sum_{k=1}^{K}{\tilde{\pi }}_{k}\log\pi_{k},\\ \; & E\left[\log q(c_{n}|y_{n},X_{n})\right]=\sum_{k=1}^{K}{\tilde{\pi }}_{k}\log{\tilde{\pi }}_{k}. \end{array}$$

## Appendix B. The Evolution of Model Structures

We demonstrate the evolutionary pathways from VAE to VaDE and from VaDE to VaDA in Figure A1. In VAE and VaDE, the sample points are assumed mutually independent. While VAE models the latent representation using the Gaussian distribution, VaDE introduces further the latent class variable to realize clustering analysis. Therefore, the latent space of VaDE forms as a Gaussian Mixture Model. VaDA is a model for longitudinal data, where we consider the dependence relationships between observations. There are two modifications from the VaDE model. Firstly, the observations belonging to a subject share the same class label. Meanwhile, they are unified through a longitudinal neural network structure to produce the outcomes.

Differences between the ELBOs of standard VAE, VaDE and VaDA are illustrated in Table A1.

**Table A1:** Comparison between the ELBOs of standard VAE, VaDE and VaDA.

Model	Reconstruction Term	KL Divergence
VAE	$\sum_{l=1}^{L}E_{q(z_{nl}|x_{nl})}\left[\log p(x_{nl}|z_{nl})\right]$	$\sum_{l=1}^{L}D_{\mathrm{KL}}\left[q\left(z_{nl}|x_{nl}\right)\left|\right|p\left(z_{nl}\right)\right]$
VaDE	$\sum_{l=1}^{L}E_{q(z_{nl}|x_{nl})}\left[\log p(x_{nl}|z_{nl})\right]$	$\sum_{l=1}^{L}D_{\mathrm{KL}}\left[q\left(z_{nl},c_{nl}|x_{nl}\right)\left|\right|p\left(z_{nl},c_{nl}\right)\right]$
VaDA	$\sum_{l=1}^{L}E_{q(z_{nl}|x_{nl})}\left[\log p(x_{nl}|z_{nl})\right]+E_{q(u_{n}|y_{n})}\left[\log p(y_{n}|u_{n})\right]$	$D_{\mathrm{KL}}\left[q\left(u_{n},Z_{n},c_{n}|y_{n},X_{n}\right)\left|\right|p\left(u_{n},Z_{n},c_{n}\right)\right]$

## Appendix C. Generating Process of Synthetic Data

Herein, we provide details on the generation of synthetic longitudinal data used in our experiments. The whole process is very similar to the generative process assumed by the proposed VaDA. We first illustrate how the model parameters were settled:

- Let $\pi_{k}=\frac{1}{K}$ for k=1,2,…,K.
- Sample $\mu_{k,d}∼U\left(-25,25\right)$ and $\sigma_{k,d}^{2}$ random positive value (The diagonal covariance matrix $\sigma_{k}^{2}$ with diagonal elements $\sigma_{k,d}^{2},d=1,2,\ldots ,D$ was generated using make_spd_matrix(D)∗np.eye(D) (in Python), where the function make_spd_matrix() is from the “scikit-learn” module and np.eye() is a function in “numpy”.) for d=1,2,…,D and k=1,2,…,K. U(a,b) is the uniform distribution.
- Sample $\beta_{k,d}∼U\left(-10,10\right)$ and $\log\sigma_{u,k}^{2}∼U\left(-0.2,0.2\right)$ for d=0,1,2,…,D and k=1,2,…,K.
- Let $f_{x}\left(z;\theta \right)=W_{2}\mathrm{RELU}\left(W_{1}\mathrm{RELU}\left(W_{0}z\right)\right)$, where $W_{0}\in R^{H_{0}\times D}$, $W_{1}\in R^{H_{1}\times H_{0}}$, $W_{2}\in R^{P\times H_{1}}$ are random matrices ($W_{0},W_{1},W_{2},G_{0},G_{1}$ and $G_{2}$ were generated using the function make_low_rank_matrix() from module “scikit-learn”). $H_{0}$ and $H_{1}$ are the numbers of units in the two hidden layers of X-decoder.
- Let $f_{y}\left(u;\phi \right)=G_{2}\mathrm{RELU}\left(G_{1}\mathrm{RELU}\left(G_{0}u\right)\right)$, where $G_{0}\in R^{Q_{0}\times L}$, $G_{1}\in R^{Q_{1}\times Q_{0}}$, $G_{2}\in R^{L\times Q_{1}}$ are random matrices. $Q_{0}$ and $Q_{1}$ are the numbers of units in the two hidden layers of Y-decoder.

In our experiment, we used $H_{0}=2^{9}$, $H_{1}=2^{8}$, $Q_{0}=2^{6}$ and $Q_{1}=2^{5}$. The procedures to generate the datasets of Scenarios 11, 01, 10 and 00 are summarized in Figure A2.

## Appendix D. Network Architectures

Herein, we supplement the network architectures used for the Synthetic data and the CelebA data in Figure A3 and Figure A4. “layer” is a module from “keras”, which is an API of TensorFlow. While function layers.Dense() defines a fully connected layer, layers.Conv2D() and layers.Conv2DTranspose() indicate the 2D convolutional layer and the 2D transposed convolutional layer respectively, which are the most basic layers used in computer vision. layers.LeakyReLU() defines an activation layer. “LeakyReLU” activation function improves the standard “ReLU” by preventing neurons from dying out [42]. In the X-decoder for CelebA, the activation function “sigmoid” was used to map the output value into [0, 1], as we had scaled the original image data to [0, 1]. In the Y-decoder, the activation of the last layer was also set as “sigmoid”, as the outcome of CelebA data was binary variable.

## Appendix E. Validation Results

The 5-fold cross-validation was conducted to decide the number of epochs required when implementing the SGVB algorithms for the deep learning methods VaDA, VaDE and VAE (Figure A5 and Figure A6). For the four synthetic scenarios, we used 500 epochs in the algorithms, where the averaged validation loss was at a relatively stable state in each case. And for the CelebA longitudinal data, the setting of 100 epochs was appropriate.

## Appendix F. Influence of MC Sample Size

In the synthetic study and the real-world experiment, we have set the MC sample size for SGVB estimator as M=1 following the convention in papers of VAE [4], VaDE [26] and VaDeSC [27]. Here, a simple sensitivity analysis is conducted to test the influence of MC sample size on the performance of VaDA. Table A2 and Table A3 show the clustering and prediction results (on testing datasets) under M∈{1, 5, 10} in Scenario 11 based on ten independent runs. As can be seen, the performance of VaDA is not very sensitive to the change of *M*. There is no apparent performance enhancement by increasing the MC sample size, which could be a result of sufficiently large batch size as mentioned in [4].

**Table A2:** Performance of VaDA evaluated by adjusted Rand index for clustering results with complete data (ARI-YX) and incomplete data (ARI-X) and by mean square error for prediction accuracy (MSE) under different MC sample size *M* with varied settings of latent dimensions *D* when the number of clusters K=3.

Metric	Samples	*D* = 2	*D* = 5	*D* = 10	*D* = 20	*D* = 100
ARI-YX	M = 1	0.963 ± 0.110	1.000 ± 0.000	0.956 ± 0.132	1.000 ± 0.000	0.873 ± 0.195
	M = 5	1.000 ± 0.000	0.958 ± 0.125	0.956 ± 0.133	0.912 ± 0.175	0.959 ± 0.122
	M = 10	1.000 ± 0.000	1.000 ± 0.000	1.000 ± 0.000	1.000 ± 0.000	0.871 ± 0.198
ARI-X	M = 1	0.963 ± 0.110	1.000 ± 0.000	0.956 ± 0.132	1.000 ± 0.000	0.873 ± 0.195
	M = 5	1.000 ± 0.000	0.958 ± 0.125	0.956 ± 0.133	0.912 ± 0.175	0.959 ± 0.122
	M = 10	1.000 ± 0.000	1.000 ± 0.000	1.000 ± 0.000	1.000 ± 0.000	0.871 ± 0.198
MSE	M = 1	0.281 ± 0.102	0.101 ± 0.029	0.067 ± 0.009	0.057 ± 0.005	0.058 ± 0.006
	M = 5	0.292 ± 0.106	0.099 ± 0.029	0.063 ± 0.008	0.059 ± 0.006	0.058 ± 0.007
	M = 10	0.323 ± 0.154	0.099 ± 0.025	0.063 ± 0.007	0.056 ± 0.004	0.058 ± 0.003

Figure A7 exhibits the training loss and validation loss of VaDA at a single run with different MC sample size *M* under the setting of K=3 and D=10.

Table A4 compares the overall memory usage and time consumption between the algorithms of VAE, VaDE and VaDA under different experimental settings in Scenario 11 over ten independent runs. The computational environment was established on the LINUX64 operating system, utilizing an Intel64 CPU architecture. All the algorithms were implemented using dual cores. The memory usage and the time consumption have an apparently increasing tendency as the MC sample size gets larger. Meanwhile, VaDA has approximately doubled memory usage and time consumption compared with VaDE. This is a result of modeling the data of outcomes, as VaDA also conducts the task of longitudinal prediction.

**Table A3:** Performance of VaDA evaluated by adjusted Rand index for clustering results with complete data (ARI-YX) and incomplete data (ARI-X) and by mean square error for prediction accuracy (MSE) under different MC sample size *M* with varied settings of the number of clusters *K* when latent dimensions D=10.

Metric	Samples	*K* = 2	*K* = 3	*K* = 4
ARI-YX	M = 1	0.574 ± 0.025	0.956 ± 0.132	1.000 ± 0.000
	M = 5	0.573 ± 0.019	0.956 ± 0.133	1.000 ± 0.000
	M = 10	0.574 ± 0.020	1.000 ± 0.000	1.000 ± 0.000
ARI-X	M = 1	0.574 ± 0.025	0.956 ± 0.132	1.000 ± 0.000
	M = 5	0.573 ± 0.019	0.956 ± 0.133	1.000 ± 0.000
	M = 10	0.574 ± 0.020	1.000 ± 0.000	1.000 ± 0.000
MSE	M = 1	0.062 ± 0.006	0.067 ± 0.009	0.065 ± 0.002
	M = 5	0.064 ± 0.005	0.063 ± 0.008	0.063 ± 0.006
	M = 10	0.068 ± 0.006	0.063 ± 0.007	0.062 ± 0.004

**Table A4:** Comparison of overall memory usage and time consumption between the algorithms of VAE, VaDE and VaDA under different experimental settings over ten independent runs.

Method	*K*	*D*	*M* = 1			*M* = 5			*M* = 10		
			CPU	Memory (GiB)	Time (m)	CPU	Memory (GiB)	Time (m)	CPU	Memory (GiB)	Time (m)
VAE	–	2	2	4.56	64.27	2	6.31	109.83	2	8.70	177.33
	–	5	2	4.54	64.33	2	6.56	110.58	2	8.60	171.15
	–	10	2	4.56	63.85	2	6.52	110.23	2	8.71	169.33
	–	20	2	4.39	66.00	2	6.38	107.62	2	8.79	168.02
	–	100	2	4.48	64.98	2	6.53	120.65	2	8.71	205.22
VaDE	3	2	2	6.71	79.03	2	8.89	116.18	2	10.85	180.60
	3	5	2	6.96	75.13	2	8.80	122.15	2	10.93	184.60
	3	10	2	6.66	78.03	2	8.75	121.20	2	10.96	188.30
	3	20	2	6.96	78.82	2	8.73	126.58	2	10.86	189.65
	3	100	2	6.75	81.17	2	8.88	134.02	2	11.08	221.92
	2	10	2	6.43	69.42	2	8.48	113.25	2	10.44	193.63
	4	10	2	7.47	78.10	2	9.16	120.43	2	11.31	198.57
VaDA	3	2	2	13.07	136.13	2	17.80	230.40	2	23.14	333.55
	3	5	2	13.26	145.47	2	17.98	227.88	2	23.31	320.53
	3	10	2	13.62	141.22	2	17.91	231.87	2	23.15	335.52
	3	20	2	13.18	145.25	2	17.70	235.70	2	23.07	342.47
	3	100	2	13.43	152.73	2	17.79	235.28	2	23.28	364.47
	2	10	2	12.16	132.18	2	16.44	219.83	2	22.08	319.65
	4	10	2	14.61	151.97	2	19.14	241.77	2	24.56	347.42

## Appendix G. Instructions of PCA

When performing the method VaDE+MM, VAE+GMM+MM and GMM+MM, we encountered frequently the problem in marginal model that “the design matrix is rank deficiency and the GEE cannot proceed”. This is a common problem in linear regression analysis with high-dimensional data, where dimension reduction or feature transformation is required. As a simple and efficient treatment, we used the PCA to make a projection of the feature (representation) data in each obtained cluster into orthogonal principal components. In a cluster, there were generally two situations to be treated: the number of observations in that cluster was larger than the dimensions of feature data; the number of observations in that cluster equaled to or was smaller than the dimensions. In the first situation, the traditional PCA was applied and the original dimensions of data was kept. In the second situation, the traditional PCA could not proceed, we had to use the sparse PCA [31], where sparse loadings were obtained by imposing the 1-norm constraint on the regression coefficients from viewing the PCA as a regression-type optimization problem. The number of principal components was set as $N_{k}-1$, where $N_{k}$ was the number of observations in cluster *k*. Throughout the experimental study, the marginal models were fitted using the R package “geepack”. The remedies by sparse PCA were implemented using the R package “elasticnet”.
