# Supplementary material for: Variational Deep Alliance: A Generative Auto-Encoding Approach to Longitudinal Data Analysis
Source: Entropy (Basel). 2026 Jan 18;28(1):113. doi: 10.3390/e28010113 (PMC12840063; doi:10.3390/e28010113)
Supplement: Supplementary file 1 [file entropy-28-00113-s001.zip › entropy-4063623-supplementary.pdf]

Supplementary Materials: Variational Deep Alliance: A Generative Auto-encoding Approach to Longitudinal Data Analysis

Shan Feng *, Wenxian Xie and Yufeng Nie *

S1. Training data results in synthetic data experiments

Table S1. Prediction results on the training datasets evaluated by the mean square error with varied settings of the latent dimensions D when the number of clusters $K = 3$.

Scenario	Method	$D = 2$	$D = 5$	$D = 10$	$D = 20$	$D = 100$
11	VaDA	0.283 ± 0.123	0.124 ± 0.047	0.068 ± 0.006	0.058 ± 0.004	0.061 ± 0.008
	VaDE+MM0	0.766 ± 0.013	0.745 ± 0.012	0.724 ± 0.006	0.720 ± 0.005	0.708 ± 0.006
	VaDE+MM1	0.769 ± 0.013	0.748 ± 0.012	0.727 ± 0.006	0.724 ± 0.005	0.716 ± 0.006
	VaDE+MM2	0.771 ± 0.014	0.747 ± 0.013	0.726 ± 0.006	0.723 ± 0.005	0.723 ± 0.005
	VAE+GMM+MM0	0.722 ± 0.005	0.722 ± 0.006	0.721 ± 0.005	0.720 ± 0.005	0.710 ± 0.007
	VAE+GMM+MM1	0.726 ± 0.005	0.726 ± 0.006	0.725 ± 0.005	0.724 ± 0.005	0.718 ± 0.006
	VAE+GMM+MM2	0.723 ± 0.005	0.724 ± 0.006	0.723 ± 0.005	0.723 ± 0.005	0.723 ± 0.005
	GMM+MM0	—	—	—	—	0.707 ± 0.008
	GMM+MM1	—	—	—	—	0.716 ± 0.006
	GMM+MM2	—	—	—	—	0.723 ± 0.005
01	VaDA	0.225 ± 0.087	0.072 ± 0.039	0.052 ± 0.006	0.048 ± 0.009	0.050 ± 0.012
	VaDE+MM0	0.393 ± 0.052	0.263 ± 0.065	0.078 ± 0.044	0.006 ± 0.003	0.004 ± 0.000
	VaDE+MM1	0.782 ± 0.136	0.462 ± 0.198	0.109 ± 0.087	0.006 ± 0.004	0.004 ± 0.000
	VaDE+MM2	0.782 ± 0.137	0.531 ± 0.218	0.110 ± 0.086	0.006 ± 0.003	0.004 ± 0.000
	VAE+GMM+MM0	0.045 ± 0.001	0.034 ± 0.001	0.014 ± 0.025	0.009 ± 0.016	0.010 ± 0.015
	VAE+GMM+MM1	0.045 ± 0.001	0.034 ± 0.001	0.014 ± 0.025	0.009 ± 0.016	0.010 ± 0.015
	VAE+GMM+MM2	0.045 ± 0.001	0.034 ± 0.001	0.014 ± 0.025	0.009 ± 0.016	0.010 ± 0.015
	GMM+MM0	—	—	—	—	0.003 ± 0.000
	GMM+MM1	—	—	—	—	0.003 ± 0.000
	GMM+MM2	—	—	—	—	0.003 ± 0.000
10	VaDA	0.092 ± 0.004	0.092 ± 0.004	0.092 ± 0.003	0.092 ± 0.003	0.091 ± 0.004
	VaDE+MM0	0.999 ± 0.007	0.998 ± 0.006	0.997 ± 0.007	0.996 ± 0.006	0.983 ± 0.009
	VaDE+MM1	1.000 ± 0.007	1.000 ± 0.006	0.999 ± 0.007	0.998 ± 0.006	0.984 ± 0.009
	VaDE+MM2	0.999 ± 0.007	0.999 ± 0.007	0.999 ± 0.007	0.999 ± 0.007	0.998 ± 0.007
	VAE+GMM+MM0	0.999 ± 0.007	0.998 ± 0.006	0.997 ± 0.007	0.995 ± 0.007	0.980 ± 0.009
	VAE+GMM+MM1	1.000 ± 0.007	0.999 ± 0.006	0.998 ± 0.007	0.996 ± 0.007	0.981 ± 0.009
	VAE+GMM+MM2	0.999 ± 0.007	0.999 ± 0.007	0.999 ± 0.007	0.999 ± 0.007	0.998 ± 0.006
	GMM+MM0	—	—	—	—	0.980 ± 0.008
	GMM+MM1	—	—	—	—	0.981 ± 0.008
	GMM+MM2	—	—	—	—	0.998 ± 0.007
00	VaDA	0.108 ± 0.007	0.109 ± 0.011	0.108 ± 0.010	0.106 ± 0.009	0.102 ± 0.002
	VaDE+MM0	0.102 ± 0.004	0.101 ± 0.008	0.097 ± 0.004	0.092 ± 0.004	0.086 ± 0.003
	VaDE+MM1	0.102 ± 0.004	0.103 ± 0.019	0.097 ± 0.004	0.092 ± 0.004	0.086 ± 0.003
	VaDE+MM2	0.102 ± 0.004	0.103 ± 0.015	0.097 ± 0.004	0.092 ± 0.004	0.086 ± 0.003
	VAE+GMM+MM0	0.100 ± 0.002	0.099 ± 0.003	0.096 ± 0.003	0.090 ± 0.004	0.088 ± 0.003
	VAE+GMM+MM1	0.100 ± 0.002	0.099 ± 0.003	0.096 ± 0.003	0.090 ± 0.004	0.088 ± 0.003
	VAE+GMM+MM2	0.100 ± 0.002	0.099 ± 0.003	0.096 ± 0.003	0.090 ± 0.004	0.088 ± 0.003
	GMM+MM0	—	—	—	—	0.012 ± 0.022
	GMM+MM1	—	—	—	—	0.012 ± 0.022
	GMM+MM2	—	—	—	—	0.012 ± 0.022

Table S2. Prediction results on the training datasets evaluated by the mean square error with varied settings of the number of clusters K when the latent dimensions $D = 10$.

Scenario	Method	$K = 2$	$K = 3$	$K = 4$
11	VaDA	0.071 ± 0.017	0.068 ± 0.006	0.067 ± 0.007
	VaDE+MM0	0.726 ± 0.005	0.724 ± 0.006	0.727 ± 0.006
	VaDE+MM1	0.729 ± 0.005	0.727 ± 0.006	0.731 ± 0.006
	VaDE+MM2	0.727 ± 0.006	0.726 ± 0.006	0.729 ± 0.006
	VAE+GMM+MM0	0.727 ± 0.005	0.721 ± 0.005	0.720 ± 0.005
	VAE+GMM+MM1	0.731 ± 0.005	0.725 ± 0.005	0.724 ± 0.005
	VAE+GMM+MM2	0.728 ± 0.005	0.723 ± 0.005	0.723 ± 0.005
	GMM+MM0	0.712 ± 0.006	0.707 ± 0.008	0.701 ± 0.010
	GMM+MM1	0.719 ± 0.005	0.716 ± 0.006	0.712 ± 0.007
	GMM+MM2	0.723 ± 0.005	0.723 ± 0.005	0.723 ± 0.005
01	VaDA	0.052 ± 0.005	0.052 ± 0.006	0.051 ± 0.005
	VaDE+MM0	0.108 ± 0.027	0.078 ± 0.044	0.112 ± 0.039
	VaDE+MM1	0.111 ± 0.034	0.109 ± 0.087	0.139 ± 0.084
	VaDE+MM2	0.113 ± 0.035	0.110 ± 0.086	0.146 ± 0.086
	VAE+GMM+MM0	0.115 ± 0.032	0.014 ± 0.025	0.006 ± 0.000
	VAE+GMM+MM1	0.115 ± 0.032	0.014 ± 0.025	0.006 ± 0.000
	VAE+GMM+MM2	0.115 ± 0.032	0.014 ± 0.025	0.006 ± 0.000
	GMM+MM0	0.004 ± 0.000	0.003 ± 0.000	0.003 ± 0.000
	GMM+MM1	0.004 ± 0.000	0.003 ± 0.000	0.003 ± 0.000
	GMM+MM2	0.004 ± 0.000	0.003 ± 0.000	0.003 ± 0.000
10	VaDA	0.095 ± 0.011	0.092 ± 0.003	0.094 ± 0.007
	VaDE+MM0	0.998 ± 0.007	0.997 ± 0.007	0.998 ± 0.007
	VaDE+MM1	0.999 ± 0.007	0.999 ± 0.007	0.999 ± 0.007
	VaDE+MM2	0.999 ± 0.007	0.999 ± 0.007	0.999 ± 0.007
	VAE+GMM+MM0	0.998 ± 0.007	0.997 ± 0.007	0.997 ± 0.007
	VAE+GMM+MM1	0.999 ± 0.007	0.998 ± 0.007	0.998 ± 0.007
	VAE+GMM+MM2	0.999 ± 0.007	0.999 ± 0.007	0.999 ± 0.007
	GMM+MM0	0.986 ± 0.007	0.980 ± 0.008	0.981 ± 0.009
	GMM+MM1	0.987 ± 0.007	0.981 ± 0.008	0.981 ± 0.009
	GMM+MM2	0.998 ± 0.006	0.998 ± 0.007	0.998 ± 0.007
00	VaDA	0.107 ± 0.007	0.108 ± 0.010	0.109 ± 0.014
	VaDE+MM0	0.098 ± 0.002	0.097 ± 0.004	0.097 ± 0.003
	VaDE+MM1	0.098 ± 0.002	0.097 ± 0.004	0.097 ± 0.003
	VaDE+MM2	0.098 ± 0.002	0.097 ± 0.004	0.097 ± 0.003
	VAE+GMM+MM0	0.098 ± 0.002	0.096 ± 0.003	0.095 ± 0.003
	VAE+GMM+MM1	0.098 ± 0.002	0.096 ± 0.003	0.095 ± 0.003
	VAE+GMM+MM2	0.098 ± 0.002	0.096 ± 0.003	0.095 ± 0.003
	GMM+MM0	0.054 ± 0.005	0.012 ± 0.022	0.017 ± 0.024
	GMM+MM1	0.054 ± 0.005	0.012 ± 0.022	0.017 ± 0.024
	GMM+MM2	0.054 ± 0.005	0.012 ± 0.022	0.017 ± 0.024

Table S3. Clustering results on the training datasets evaluated by the adjusted Rand index with varied settings of the latent dimensions D when the number of clusters $K = 3$.

Scenario	Method	$D = 2$	$D = 5$	$D = 10$	$D = 20$	$D = 100$
11	VaDA-YX	0.986 ± 0.073	0.943 ± 0.144	0.957 ± 0.128	0.971 ± 0.108	0.914 ± 0.172
	VaDA-X	0.986 ± 0.073	0.943 ± 0.144	0.957 ± 0.128	0.971 ± 0.108	0.914 ± 0.172
	VaDE	0.370 ± 0.145	0.427 ± 0.164	0.785 ± 0.216	0.984 ± 0.081	1.000 ± 0.000
	VAE+GMM	1.000 ± 0.000	0.981 ± 0.101	1.000 ± 0.000	0.946 ± 0.165	0.935 ± 0.139
	GMM	—	—	—	—	0.816 ± 0.265
01	VaDA-YX	1.000 ± 0.000	1.000 ± 0.000	0.972 ± 0.106	0.986 ± 0.077	0.917 ± 0.167
	VaDA-X	1.000 ± 0.000	1.000 ± 0.000	0.972 ± 0.106	0.986 ± 0.077	0.917 ± 0.167
	VaDE	0.306 ± 0.158	0.409 ± 0.133	0.724 ± 0.203	1.000 ± 0.001	1.000 ± 0.000
	VAE+GMM	1.000 ± 0.000	1.000 ± 0.000	0.945 ± 0.168	0.964 ± 0.138	0.921 ± 0.172
	GMM	—	—	—	—	0.816 ± 0.265
10	VaDA-YX	0.942 ± 0.148	0.957 ± 0.128	0.957 ± 0.129	0.957 ± 0.128	0.971 ± 0.110
	VaDA-X	0.942 ± 0.148	0.957 ± 0.128	0.957 ± 0.129	0.957 ± 0.128	0.971 ± 0.110
	VaDE	0.611 ± 0.272	0.539 ± 0.247	0.587 ± 0.169	0.592 ± 0.292	0.666 ± 0.272
	VAE+GMM	0.803 ± 0.264	0.853 ± 0.247	0.816 ± 0.265	0.907 ± 0.210	0.852 ± 0.249
	GMM	—	—	—	—	0.912 ± 0.179
00	VaDA-YX	0.927 ± 0.162	0.941 ± 0.148	1.000 ± 0.000	0.986 ± 0.074	0.942 ± 0.148
	VaDA-X	0.927 ± 0.162	0.942 ± 0.148	1.000 ± 0.000	0.986 ± 0.074	0.942 ± 0.148
	VaDE	0.651 ± 0.215	0.579 ± 0.253	0.630 ± 0.212	0.651 ± 0.217	0.579 ± 0.204
	VAE+GMM	0.780 ± 0.258	0.818 ± 0.262	0.779 ± 0.275	0.742 ± 0.281	0.926 ± 0.191
	GMM	—	—	—	—	0.914 ± 0.176

Table S4. Clustering results on the training datasets evaluated by the adjusted Rand index with varied settings of the number of clusters K when the latent dimensions $D = 10$.

Scenario	Method	$K = 2$	$K = 3$	$K = 4$
11	VaDA-YX	0.570 ± 0.010	0.957 ± 0.128	0.986 ± 0.076
	VaDA-X	0.570 ± 0.010	0.957 ± 0.128	0.986 ± 0.076
	VaDE	0.546 ± 0.044	0.785 ± 0.216	0.462 ± 0.145
	VAE+GMM	0.572 ± 0.013	1.000 ± 0.000	0.872 ± 0.079
	GMM	0.571 ± 0.011	0.816 ± 0.265	0.878 ± 0.174
01	VaDA-YX	0.572 ± 0.013	0.972 ± 0.106	0.986 ± 0.073
	VaDA-X	0.572 ± 0.013	0.972 ± 0.106	0.986 ± 0.073
	VaDE	0.558 ± 0.037	0.724 ± 0.203	0.566 ± 0.150
	VAE+GMM	0.573 ± 0.010	0.945 ± 0.168	0.888 ± 0.087
	GMM	0.571 ± 0.011	0.816 ± 0.265	0.878 ± 0.174
10	VaDA-YX	0.575 ± 0.011	0.957 ± 0.129	1.000 ± 0.000
	VaDA-X	0.575 ± 0.011	0.957 ± 0.129	1.000 ± 0.000
	VaDE	0.567 ± 0.014	0.587 ± 0.169	0.618 ± 0.231
	VAE+GMM	0.575 ± 0.011	0.816 ± 0.265	0.819 ± 0.174
	GMM	0.573 ± 0.011	0.912 ± 0.179	0.873 ± 0.203
00	VaDA-YX	0.573 ± 0.012	1.000 ± 0.000	0.955 ± 0.134
	VaDA-X	0.573 ± 0.012	1.000 ± 0.000	0.955 ± 0.134
	VaDE	0.570 ± 0.011	0.630 ± 0.212	0.633 ± 0.149
	VAE+GMM	0.573 ± 0.010	0.779 ± 0.275	0.819 ± 0.178
	GMM	0.574 ± 0.011	0.914 ± 0.176	0.871 ± 0.197

S2. Image editing based on VaDE and VAE+GMM

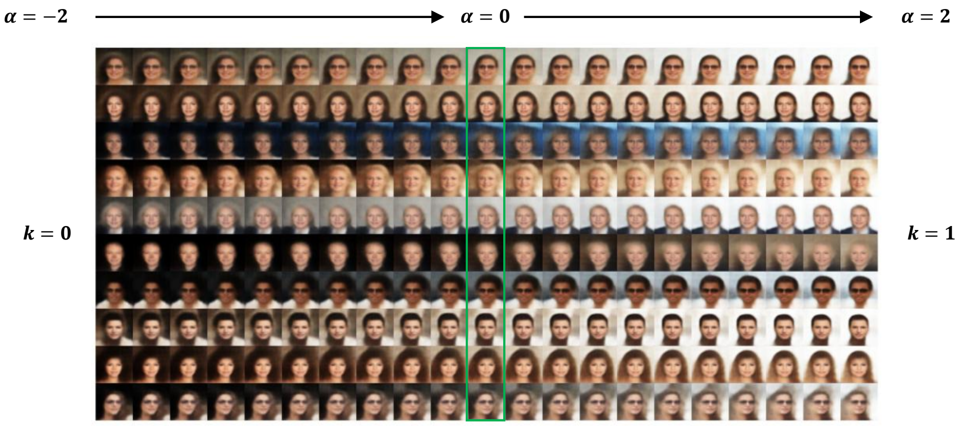

Figure S1. Image editing by concept vector between each pair of clusters based on the results of VaDE with $K = 2$. The gradient α takes value in $[-2, 2]$. The results at $\alpha = 0$ (enclosed by green box) correspond to the reconstruction of original images.

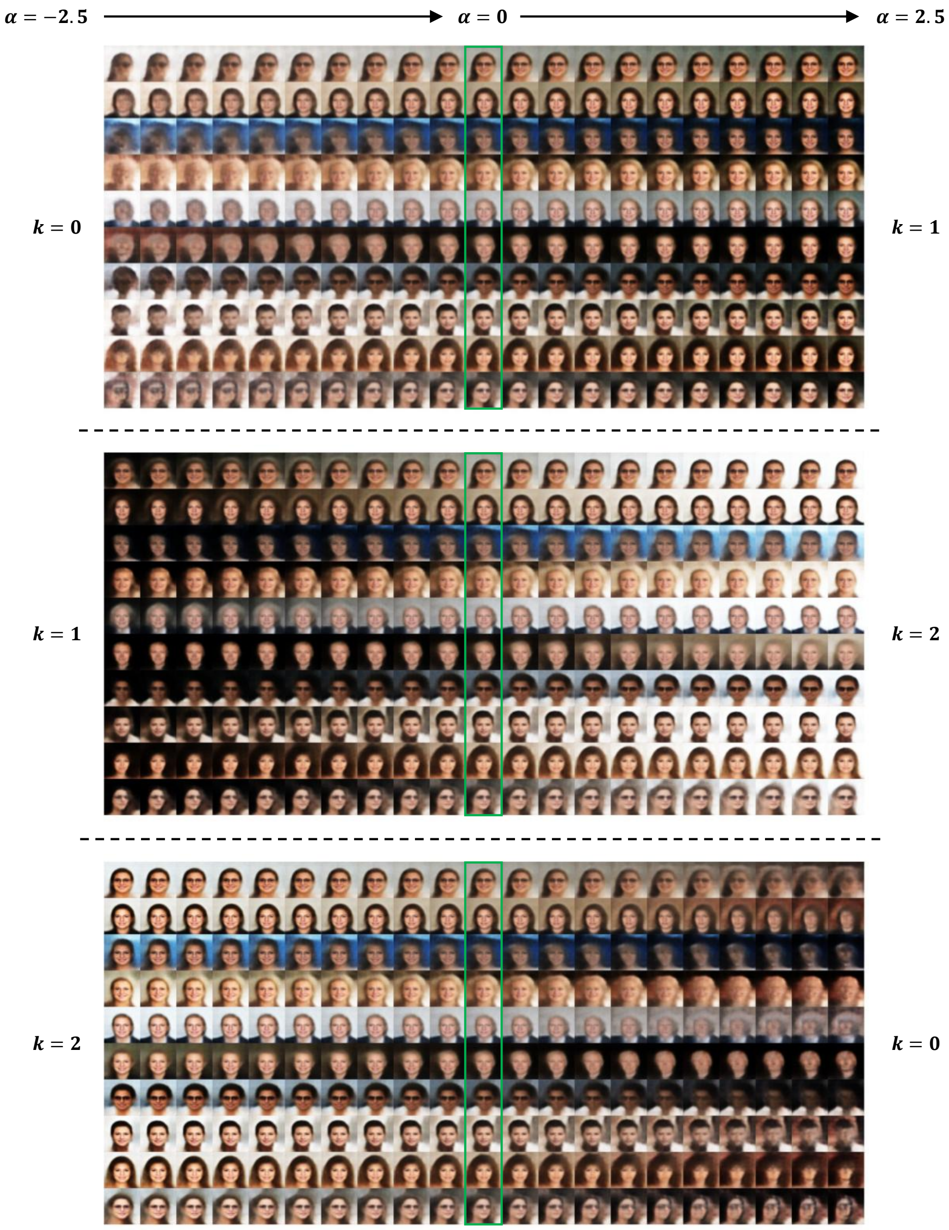

Figure S2. Image editing by concept vector between each pair of clusters based on the results of VaDE with $K = 3$. The gradient α takes value in $[-2.5, 2.5]$. The results at $\alpha = 0$ (enclosed by green box) correspond to the reconstruction of original images.

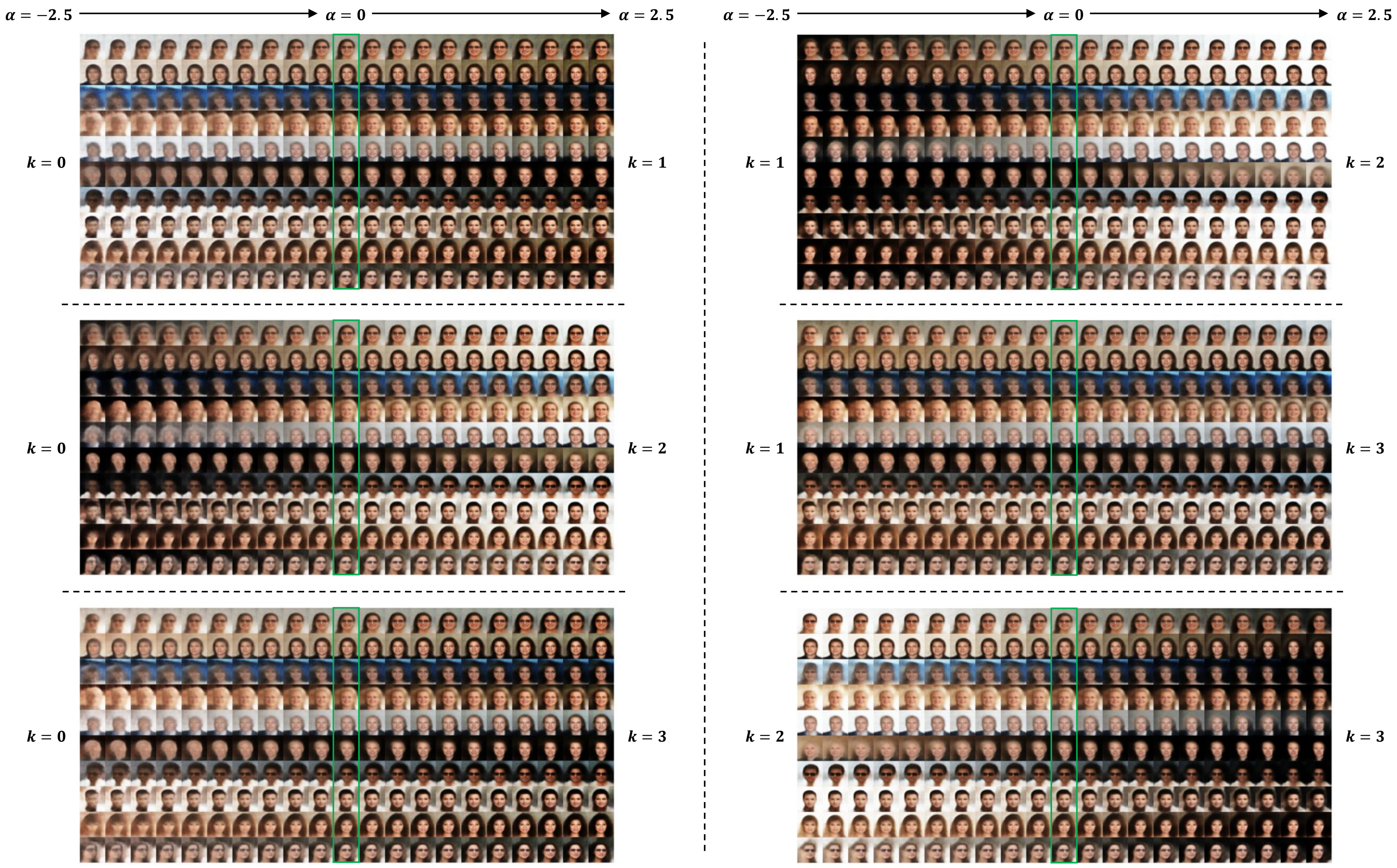

Figure S3. Image editing by concept vector between each pair of clusters based on the results of VaDE with $K = 4$. The gradient α takes value in $[-2.5, 2.5]$. The results at $\alpha = 0$ (enclosed by green box) correspond to the reconstruction of original images.

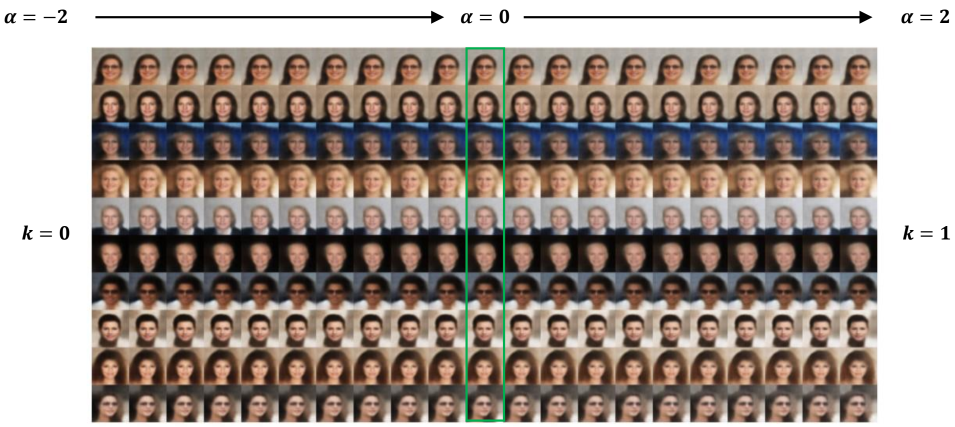

Figure S4. Image editing by concept vector between each pair of clusters based on the results of VAE+GMM with $K = 2$. The gradient α takes value in $[-2, 2]$. The results at $\alpha = 0$ (enclosed by green box) correspond to the reconstruction of original images.

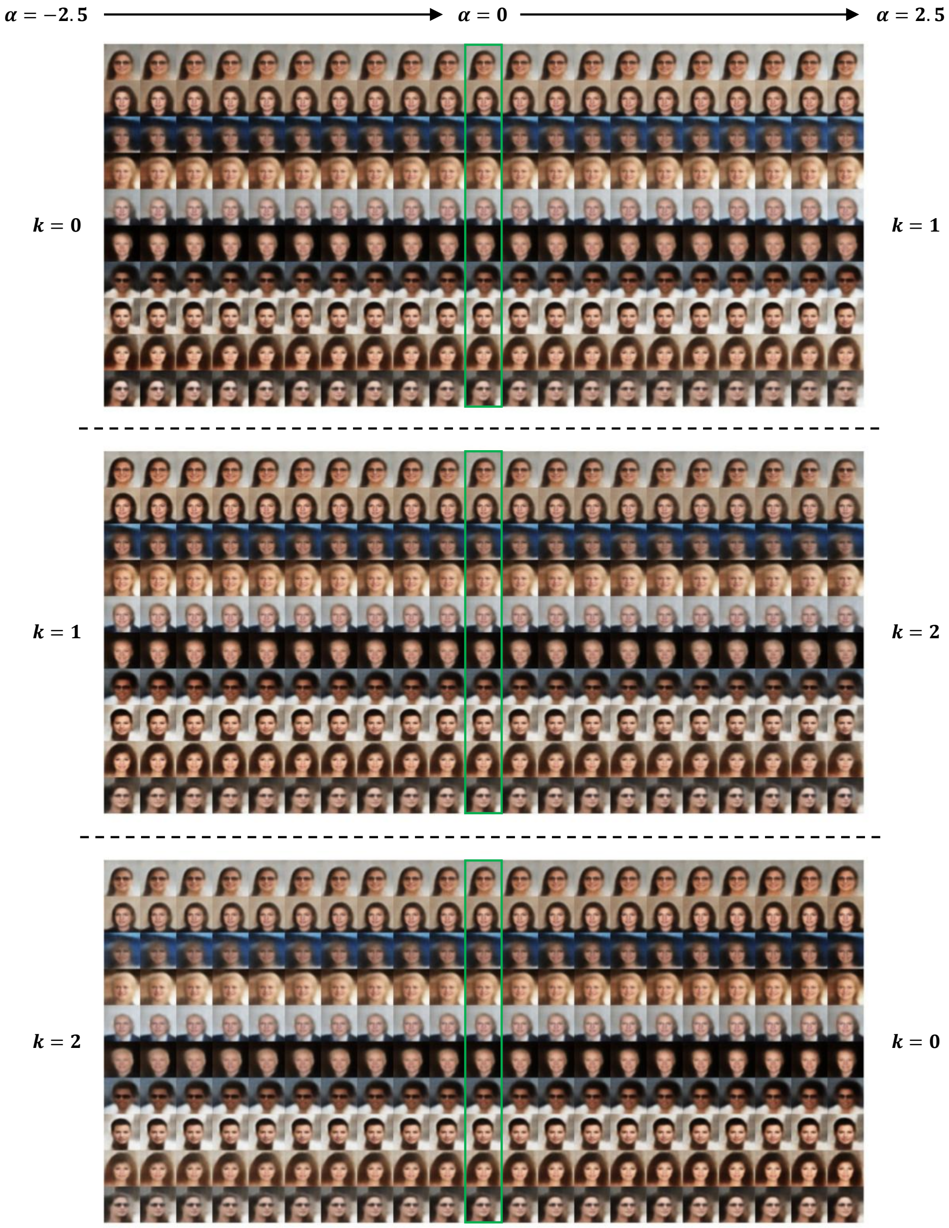

Figure S5. Image editing by concept vector between each pair of clusters based on the results of VAE+GMM with $K = 3$. The gradient α takes value in $[-2.5, 2.5]$. The results at $\alpha = 0$ (enclosed by green box) correspond to the reconstruction of original images.

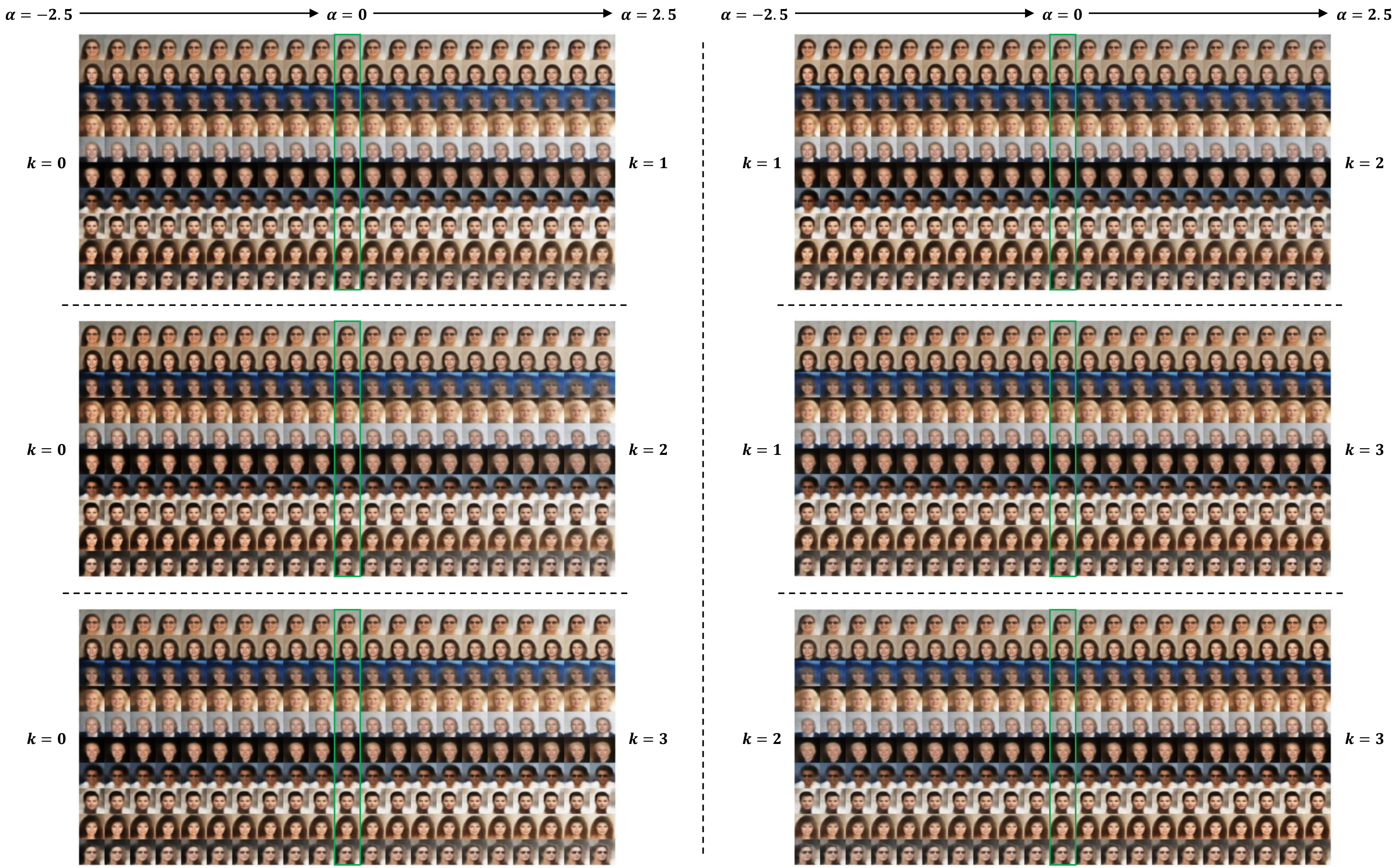

Figure S6. Image editing by concept vector between each pair of clusters based on the results of VAE+GMM with $K = 4$. The gradient α takes value in $[-2.5, 2.5]$. The results at $\alpha = 0$ (enclosed by green box) correspond to the reconstruction of original images.

S3. A pilot study on misspecification of the network structures

Herein, we present the results of a pilot study for investigating the impacts of misspecification of the network structures in VaDA. While the main paper presents a broad synthetic study, we narrow our focus here to Scenario 11 to provide a detailed examination. The hyperparameters K and D were fixed at their true values, i.e. $K = 3$ and $D = 10$. Firstly, we considered the encoder and decoder network architectures with redundancy (Figure S7), which as expected could induce overfitting problems. Therefore, we also considered the architectures with "dropout" layers [1] added (Figure S8).

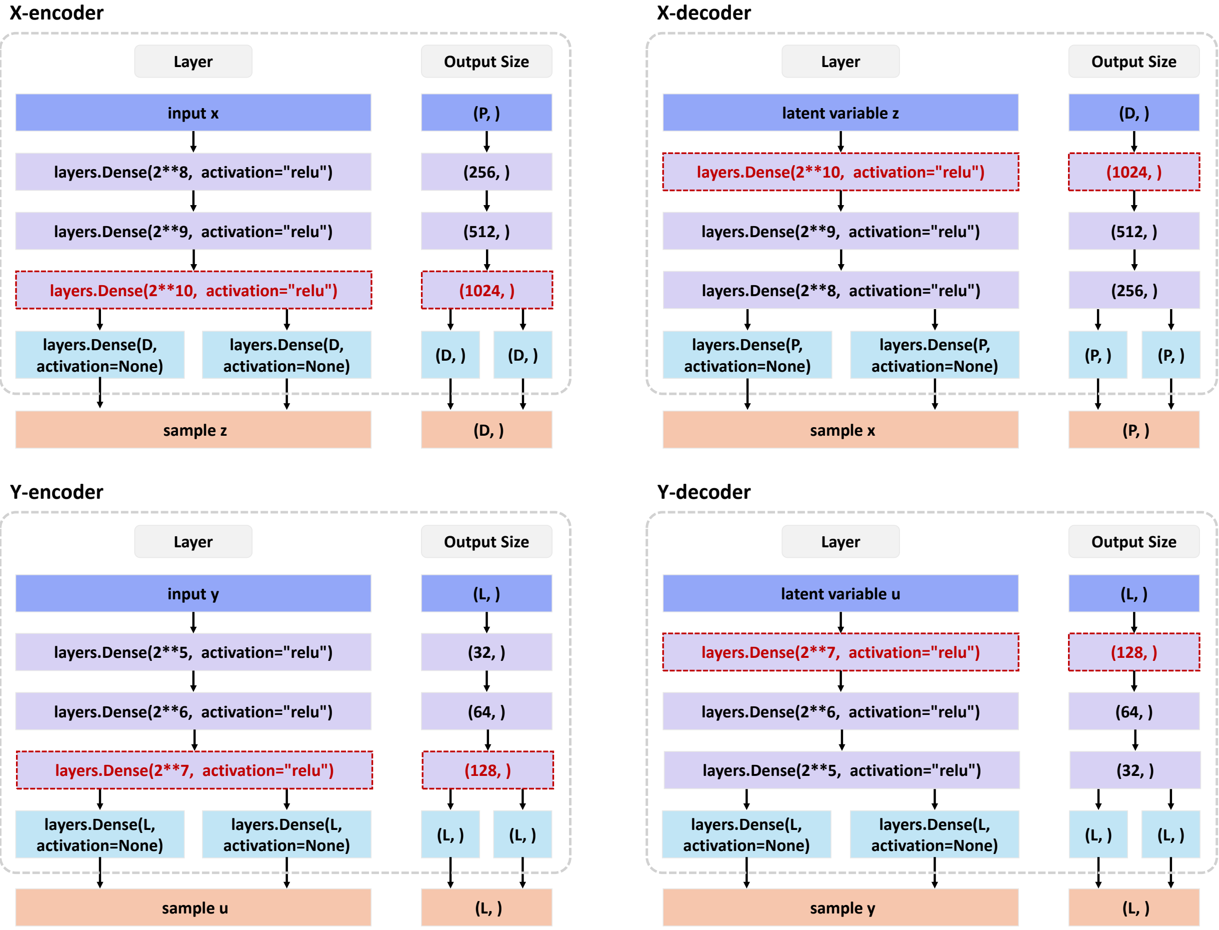

Figure S7. Architectures of encoder and decoder networks in VaDA for synthetic data with redundancy.

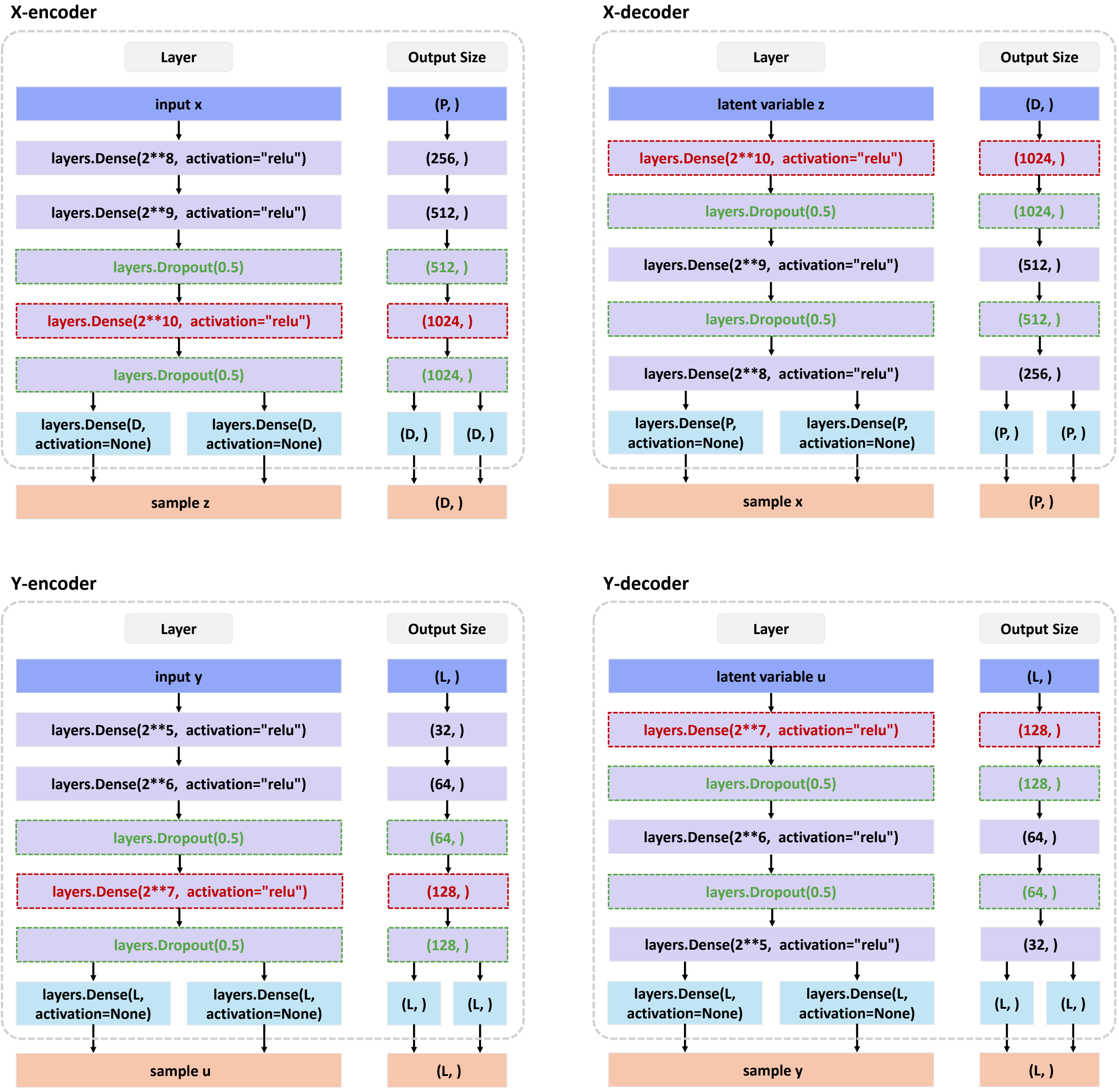

Figure S8. Architectures of encoder and decoder networks in VaDA for synthetic data with dropout.

Table S5 compares the performance of VaDA under different network structures (the results were obtained across ten replications). The normal one is what we have used in the main experiment similar to the structure in data generating process. As the results show, the redundancy degrades the prediction accuracy of VaDA, and incorporating “dropout” layers mitigates this issue, resulting in lower variance predictions. In addition, it is interesting to find that the redundancy improves the clustering performance of VaDA and the “dropout” technique has little further impact on the results.

Table S5. The performance of VaDA with normal, redundant and dropout included network structures evaluated on the metrics of adjusted Rand index for clustering results from VaDA-YX (ARI-YX) and from VaDA-X (ARI-X), mean square error for prediction accuracy (MSE).

Training			
Metric	Normal	Redundant	Dropout
ARI-YX	0.829 ± 0.210	1.000 ± 0.000	1.000 ± 0.000
ARI-X	0.829 ± 0.210	1.000 ± 0.001	1.000 ± 0.000
MSE	0.065 ± 0.009	0.153 ± 0.052	0.120 ± 0.006
Testing			
Metric	Normal	Redundant	Dropout
ARI-YX	0.824 ± 0.217	1.000 ± 0.000	1.000 ± 0.000
ARI-X	0.824 ± 0.217	0.999 ± 0.002	1.000 ± 0.000
MSE	0.064 ± 0.007	0.151 ± 0.066	0.119 ± 0.006

References

1. Gal, Y.; Ghahramani, Z. Dropout as a Bayesian approximation: representing model uncertainty in deep learning. In Proceedings of the the 33rd International Conference on Machine Learning, New York, USA, June 2016; Vol. 48.

Disclaimer/Publisher's Note: The statements, opinions and data contained in all publications are solely those of the individual author(s) and contributor(s) and not of MDPI and/or the editor(s). MDPI and/or the editor(s) disclaim responsibility for any injury to people or property resulting from any ideas, methods, instructions or products referred to in the content.
